# Comprehensive Functional Annotation of Seventy-One Breast Cancer Risk Loci

**DOI:** 10.1371/journal.pone.0063925

**Published:** 2013-05-22

**Authors:** Suhn Kyong Rhie, Simon G. Coetzee, Houtan Noushmehr, Chunli Yan, Jae Mun Kim, Christopher A. Haiman, Gerhard A. Coetzee

**Affiliations:** 1 Department of Preventive Medicine, Keck School of Medicine, University of Southern California, Los Angeles, California, United States of America; 2 Norris Cancer Center, Keck School of Medicine, University of Southern California, Los Angeles, California, United States of America; 3 Zilkha Neurogenetic Institute, Keck School of Medicine, University of Southern California, Los Angeles, California, United States of America; 4 Department of Urology, Keck School of Medicine, University of Southern California, Los Angeles, California, United States of America; Vanderbilt University Medical Center, United States of America

## Abstract

Breast Cancer (BCa) genome-wide association studies revealed allelic frequency differences between cases and controls at index single nucleotide polymorphisms (SNPs). To date, 71 loci have thus been identified and replicated. More than 320,000 SNPs at these loci define BCa risk due to linkage disequilibrium (LD). We propose that BCa risk resides in a subgroup of SNPs that functionally affects breast biology. Such a shortlist will aid in framing hypotheses to prioritize a manageable number of likely disease-causing SNPs. We extracted all the SNPs, residing in 1 Mb windows around breast cancer risk index SNP from the 1000 genomes project to find correlated SNPs. We used FunciSNP, an R/Bioconductor package developed in-house, to identify potentially functional SNPs at 71 risk loci by coinciding them with chromatin biofeatures. We identified 1,005 SNPs in LD with the index SNPs (r^2^≥0.5) in three categories; 21 in exons of 18 genes, 76 in transcription start site (TSS) regions of 25 genes, and 921 in enhancers. Thirteen SNPs were found in more than one category. We found two correlated and predicted non-benign coding variants (rs8100241 in exon 2 and rs8108174 in exon 3) of the gene, ANKLE1. Most putative functional LD SNPs, however, were found in either epigenetically defined enhancers or in gene TSS regions. Fifty-five percent of these non-coding SNPs are likely functional, since they affect response element (RE) sequences of transcription factors. Functionality of these SNPs was assessed by expression quantitative trait loci (eQTL) analysis and allele-specific enhancer assays. Unbiased analyses of SNPs at BCa risk loci revealed new and overlooked mechanisms that may affect risk of the disease, thereby providing a valuable resource for follow-up studies.

## Introduction

Apart from a few examples of genetic mutations with high penetrance, such as found in *BRCA1* & *2* genes [1], most genetic risk of breast cancer (BCa) resides at multiple low penetrance loci, more recently identified by genome-wide association studies (GWASs) [2]. In general, GWASs utilize single nucleotide polymorphisms (SNPs) to tag common genetic variation in linkage disequilibrium (LD) blocks in order to identify genome-wide risk loci for complex diseases. To date, 71 replicated and independent BCa risk loci have been identified [3], [4], [5], [6], [7], [8], [9], [10], [11], [12], [13], [14], [15], [16]. There are thousands of SNPs in each LD block, and many of these SNPs are candidates to exert functionality in BCa risk. At the 71 BCa risk loci, at least 320,000 SNPs are associated with BCa risk. Due to this plethora of SNPs in LD, much of the heritability of complex diseases, such as BCa, remains unknown [17]. Identification of underlying mechanisms that explain how SNPs affect risk will provide a better understanding of the genetic risk of complex diseases, such as breast cancer, which is described in this study.

In contrast to Mendelian disorders, where most disease-causing mutations result in absent or non-function proteins, many complex disease-associated variants, such as for BCa are mainly found in non-coding regions of the genome. Since >90% of the genome is non-coding and risk mechanisms of complex diseases are likely due to subtle regulation of gene expression, risk-SNPs are more often found in non-coding regions. Knowledge of the non-coding regions is rudimentary compared to the protein coding part. However, recent ENCODE data dramatically demonstrated that the non-coding part of the genome is much more than simply ‘junk’ DNA and contains well-demarcated gene regulatory regions, in particular enhancers [18].

We have recently formulated a roadmap to address the functionality of risk SNPs in non-coding regions by characterizing gene regulatory regions with nucleosome and transcription factor occupancy and histone modifications [19]. Moreover, several research groups annotated genomic regions (coding and non-coding) to identify candidate functional SNPs involved in complex diseases [20], [21], [22], [23], [24], [25], [26]. However, as more next generation sequencing (NGS) data (of chromatin annotations from consortia such as ENCODE), more loci (from meta and primary GWASs), and more SNPs at ever lower minor allele frequencies (from the 1000 genomes project) become available, further analyses utilizing updated data and methods are needed for specific diseases such as BCa.

In the present study, we addressed the hypothesis that BCa risk SNPs reside in functional genomic regions such as coding exons, TSS regions, and enhancers. In order to identify potentially functional SNPs, we conducted a comprehensive analysis on 656,895 SNPs from the 1000 genomes project data released in May 2012, at the 71 BCa risk loci by measuring LD and annotating them with 11 NGS datasets, all in primary breast epithelial cells. Thus, we found 1,005 potentially functional high LD SNPs. From these, we were able to frame specific hypotheses involving 547 SNPs in terms of novel biological mechanisms; 2 SNPs were at non-benign codon changes in one gene, 42 and 503 SNPs were within response elements of known transcription factors in TSS regions and enhancers, respectively. This shortlist of potentially functional SNPs will not only aid in prioritizing a manageable number of likely functional SNPs, but also reveal hidden biological mechanisms for the etiology of breast cancer.

## Results and Discussion

### One-thousand-and-five Potentially Functional High LD SNPs in Seventy-one Breast Cancer Risk Loci

**Figure 1:**
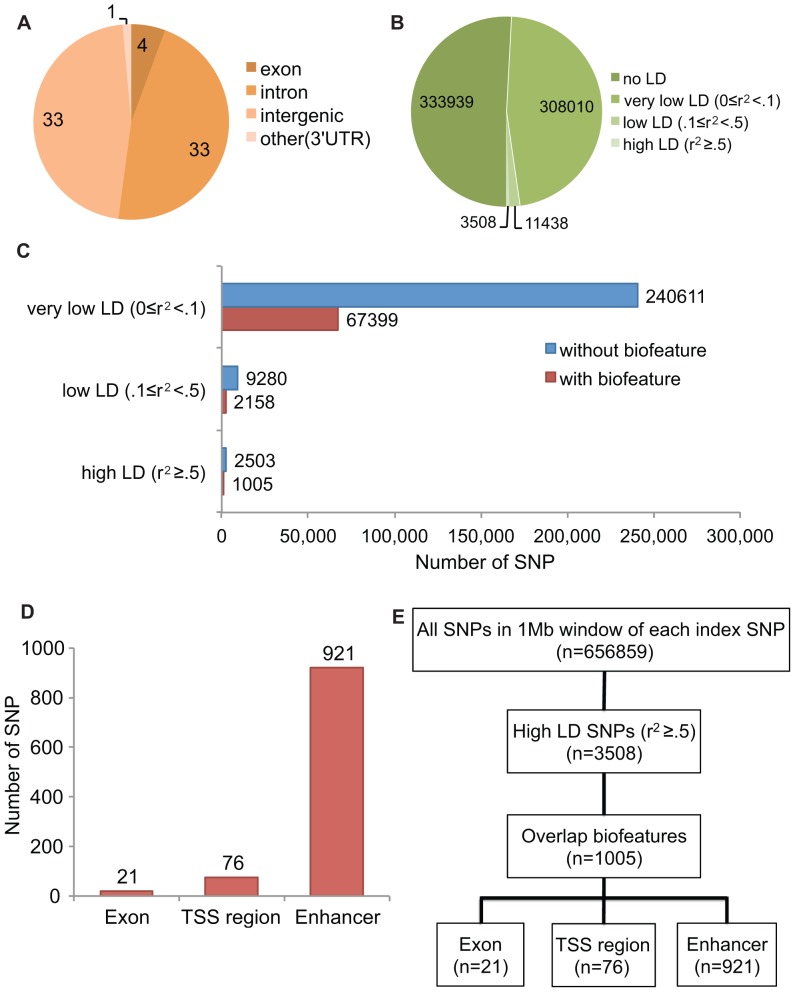
Identification of potential functional SNPs in 71 Breast cancer risk loci. (A) Genomic distribution of 71 replicated index SNPs for breast cancer risk loci. (B) SNPs residing in 1 MB windows around breast cancer risk index SNPs were categorized into the indicated four different groups by measuring LD in EUR ethnic groups. (C) SNPs in each LD group were further analyzed by their locations coinciding with biofeatures. (D) High LD SNPs within biofeatures were categorized to three groups; exon, TSS region, and enhancers. (E) The entire process was summarized in a flow diagram.

To date, 71 replicated risk loci for BCa have been identified primarily using GWASs [3], [4], [5], [6], [7], [8], [9], [10], [11], [12], [13], [14], [15], [16]. The index SNPs identified by GWASs occur mainly in non-coding DNA (33 intergenic, 33 in introns, 1 in a 3′UTR) and only 4 in coding exons (Fig. 1A, Table S1). Although index SNPs such as rs11571833 (Lys3326Term in *BRCA2* gene) [15] seem to be involved in known genetic mechanism of breast cancer tumorigenesis [1], the mechanisms for most of the other index SNPs are hidden. Additionally, these index SNPs are most likely surrogates of many other SNPs in LD, since most of the GWAS arrays were designed based on the Hapmap data to capture a large fraction of common genetic variation [27]. When we extracted SNP data for Europeans from the 1000 genomes project released in May 2012 [28], we found 308,010 very low LD (0≤r^2^<0.1), 11,438 low LD (0.1≤r^2^<0.5), and 3,508 high LD (r^2^≥0.5) SNPs at the 71 BCa risk loci (in a 1 MB window surrounding each index SNP) (Fig. 1B).

In order to identify potentially functional SNPs, we hypothesized that risk SNPs occur at sites with functionality of some form or another. Candidates are in coding exons, regulatory regions near TSS (TSS regions), and enhancers. To assist in assigning potential functionality, we performed a FunciSNP (Functional Integration of SNPs) analysis [29]. FunciSNP is an R/Bioconductor package developed in-house to evaluate positional overlap between correlated SNPs at any disease or trait locus, and available chromatin biofeatures. Here, we chose exons, TSS regions (including promoters), and enhancers as biofeatures to annotate the genome comprehensively.

Coding exon data were downloaded from the UCSC genome table browser [30]. TSS regions were defined as 3 kb windows centered on the annotated transcription start sites of genes including one or more of the following biofeatures, all in human mammary epithelial cells (HMEC): nucleosome depletion [DNase1-sensitivity and/or Formaldehyde-Assisted Isolation of Regulatory Elements (FAIRE) signals] and/or histone modifications as diagnostics of promoters (H3K4me3, H3K4me2, H3K9ac and/or H3K27ac) [31], [32], [33]. Enhancers were defined as regions in introns and intergenic regions (>1.5 kb from TSS) in HMEC, containing one or more of the following biofeatures: nucleosome depletion (DNase1-sensitivity and/or FAIRE signals) and/or histone modifications as diagnostics of enhancers (H3K4me1, H3K4me2, H3K9ac and/or H3K27ac) [31], [32], [33].

In order to identify correlated risk SNPs, a FunciSNP evaluation of each index SNP was applied by extracting all known SNPs from the 1000 genomes project database (1 Mb windows, spanning each index SNP) [28]. Biofeatures were then aligned with the positions of all curated SNPs at each region. Each SNP that overlaps with a biofeature was used to calculate the r^2^ and distance to the associated index SNP. Among 322,954 correlated SNPs (r^2^>0), 22 percent were at biofeatures (Fig. 1C). Several issues may be considered to define risk SNPs in LD. One is that low LD SNPs may be the functional risk SNP, poorly measured by the index SNP. On the other hand, high LD SNPs are more likely to be the risk SNP, since this is based on the hypothesis that the underlying functional alleles are common. We identified 1,005 SNPs in relatively high LD (r^2^≥0.5); 21 in exons, 76 in TSS regions, and 921 in enhancers (Fig. 1D) at 60 of the 71 BCa risk loci. The selection process of potentially functional variants is summarized in Fig. 1E.

### Twenty-one High LD SNPs in Exons: Two Non-benign Coding Variants in the *ANKLE1* Gene

**Figure 2:**
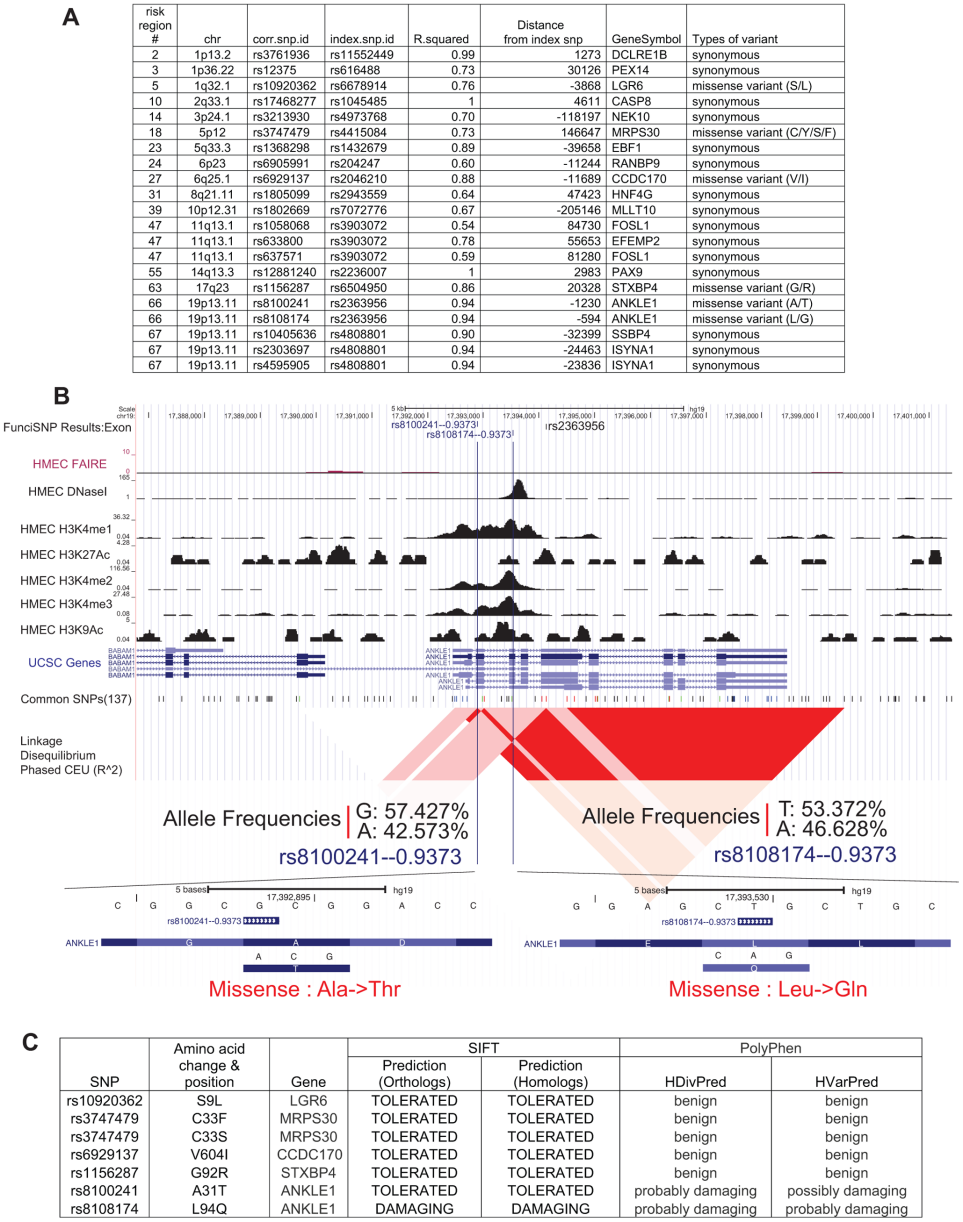
21 High LD SNPs in exon and effect of each variant to the respective protein. (A) The list of high LD SNPs (r^2^≥0.5) in exons. The risk region number was derived from Table S1 and ordered by chromosome number. Index SNP of each corrSNP and the value of r^2^ between these SNPs were listed. The distance from the index SNP to each corrSNP was shown along with the name of the nearest gene. The type of each exon variant was also annotated. (B) A genomic browser view of two high LD SNPs, rs8100241 and rs8108174. The first track showed FunciSNP results for the exons. The name of the correlated SNP (rsnumber – r^2^ value) was shown in blue. The index SNP was shown in black. The bottom tracks were biofeature tracks, RefSeq genes/mRNA/Pseudogene tracks from UCSC Genes, common SNPs (version 137), and Linkage Disequilibrium (LD) blocks. LD block, which was measured by r^2^ value in phased CEU is shown. Allele frequencies of each SNP (in all populations) and zoomed in view of the genome browser for each SNP were shown, including the amino acid changes by missense variants. (C) The effect of amino acid changes by missense variants of the respective protein was predicted by SIFT and PolyPhen [34], [35].

Twenty-one high LD SNPs (r^2^≥0.5) were annotated in exons (Fig. 2A). The majority (fifteen) results in synonymous variants. Among the six missense variants, 2 variants: rs8100241 and rs8108174 (both in the gene *ANKLE1* at locus 19p13) (Fig. 2B), are predicted to result in a non-benign change as revealed by SIFT and PolyPhen protein function prediction software [34], [35] (Fig. 2C, Table S2). The first of these is in exon 2 (causing A31T) and the other in exon 3 (causing L94Q). Both SNPs are equally and highly correlated (r^2^ = 0.94) with the original GWAS index SNP, rs2363956, which in turn also results in another non-benign amino acid change (L184W) in exon 5 of *ANKLE1* as revealed by PolyPhen analysis (Table S3). Thus, the three SNPs collectively result in two main haplotypes, which in turn create two main protein isoforms, A - L - L and T - Q - W (Fig. S1) with most likely functional consequences as revealed by SIFT and PolyPhen analyses. *ANKLE1* is expressed in breast epithelial cells [36], [37] (Fig. S2). It contains an ankyrin repeat likely involved in protein-protein interactions. Also, it is an evolutionary conserved non-membrane-bound LEM protein that shuttles between the nucleus/cytoplasm and has an enzymatically active GIY-YIG endonuclease domain [36]. This multifunctional protein has the potential of affecting many cellular phenotypes and thus cancer risk. The two allelic variants need to be modeled in protein structure-function assays to precisely determine the risk mechanisms involving them. A final interesting genomic feature of the two correlated SNPs is that their locations appear to have histone H3K4me1, -me2 and -me3 signals (Fig. 2B), pointing to possible additional potential roles in regulatory components that in turn may affect expression levels of *ANKLE1* and/or the other nearby gene, *BABAM1*. Such multi-functional SNPs will add to the complexity of BCa disease risk. Interestingly, the same locus was identified in a GWAS of ovarian cancer [38], indicating that *ANKLE1* may be generally involved in women cancers, perhaps via hormonal-mediated mechanisms.

### Seventy-six High LD SNPs in TSS Regions

Next, we studied 76 high LD SNPs, which resided at TSS regions of 25 genes (Table S4). Fifty-two percent of these genes are not only expressed in breast tissues, but their expression levels are changed during breast carcinogenesis [39], [40], [41], [42], [43], [44], [45] (Table S5). The TSS regions were defined as containing not only proximal promoters but also distal ones and perhaps also close-by (proximal) enhancers in the 3 kb windows centered at annotated TSS. These genomic regions are likely involved in gene expression regulation of the gene, primarily by altering transcription factor (TF) binding. There are approximately 2,600 proteins in the human genome that bind to DNA [46], and recently, a large number of ChIP-seq datasets were published involving many TFs [18]. However, due to the availability of a limited number of good antibodies and the requirement of high numbers of cells for ChIP assays, ChIP data are often biased towards a subgroup of TFs. As a more broader approach, we performed *in silico* searches of finding TF REs by utilizing 4 different softwares: HOMER (ChIP-seq known motifs), FIMO, Genome Trax (ChIP-seq TFBS), Haploreg (TRANSFAC, JASPAR, and PBM) [47], [48], [49], [50]. In this way, we established datasets that contain thousands of TF motifs. Among the 76 high LD SNPs in TSS regions, 42 likely affect known transcription factor binding by altering their REs as revealed by our analyses. These SNPs were located at 82 different TF motifs’ REs (Table S6). We ranked the TFs by the number of SNPs affecting their REs across the risk loci, and noted the top 10 motifs, defined as containing 2 or more SNPs affected the motifs in question (Table S7). The top motif was for Specificity Protein 1 (SP1) followed by the motif for Early Growth Response 1 (EGR1). REs of SP1 were affected at 6 TSS regional SNPs from 5 risk loci, and its binding was likely altered by the SNP alleles (Table S6). SP1 is known to be involved in many cellular processes including cell differentiation, cell growth, apoptosis, response to DNA damage, and chromatin remodeling, and its expression is up-regulated in breast cancer cells [51]. Therefore, it is reasonable to suggest that the perturbed REs by our newly identified risk SNPs may alter the binding activity of SP1 and thereby change the expression patterns of the genes, regulated by SP1.

**Figure 3:**
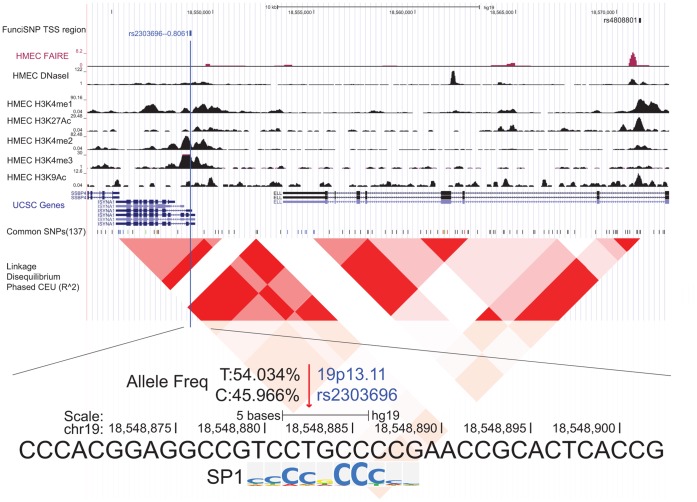
An example of TSS regional SNPs, rs2303696, in the promoter region of *ISYNA1*. The genomic browser view was shown of a TSS regional high LD SNP, rs2303696. First track shows FunciSNP results for TSS region. The name of correlated SNP (rsnumber – r^2^ value) was shown and color-coded to indicate the number of biofeatures (Fig. S3A). The index SNP was shown in black. The bottom tracks were biofeature tracks, RefSeq genes/mRNA/Pseudogene tracks from UCSC Genes, common SNPs (version 137), and Linkage Disequilibrium (LD) blocks. LD block, which was measured by r^2^ value in phased CEU is shown. Allele frequencies of rs2303696 (in all populations) and the location of this SNP in SP1 RE were shown.

One example of a TSS regional SNP is rs2303696 (at 19p13.11 risk locus), which likely alters a SP1 RE. This SNP is highly correlated (r^2^ = 0.81) with a known index SNP, rs1353747, which is located 22 kb downstream from it. The correlated SNP is located in the promoter region of Inositol-3-phosphate synthase 1 (ISYNA1) gene, which catalyzes the *de novo* synthesis of myoinositol 1-phosphate from glucose 6-phosphate (Fig. 3, Fig. S3A). Seelan et al [52] reported that E2F1 and SP1 interaction at *ISYNA1* gene promoter regulates *ISYNA1* expression level. Additionally, it is expressed in breast tissue and decreases 5–6 fold during invasive breast carcinogenesis (Table S5) [39], [45]. We propose here that the SNP may influence the regulatory activity of this gene’s promoter and thus influencing risk.

Additionally, expression quantitative trait locus (eQTL) analyses were performed to examine whether these TSS regional SNPs are associated with messenger RNA (mRNA) level by using publicly available datasets [53], [54], [55], [56], [57], [58], [59], [60], [61] (Table S8). Among 76 high LD SNPs in TSS regions, 30 SNPs are significantly associated with nearby gene mRNA level (P<10^−5^). As an example, rs832552 (at *MAP3K1* promoter region) changes the expression level of C5orf35 gene in estrogen receptor positive breast cancer tissues as its allele changes (Table 1).

**Table 1:** eQTL analyses on high LD SNPs in breast cancer cells.

Index SNP	High LD SNP	r^2^	Target Gene	eQTL P-value	Cell type	Reference
rs889312	rs832552	0.61	*C5orf35*	2.46e-6	Estrogen receptor positive breast cancer	(Li et al., 2013) [53]
rs889312	rs252913	0.59	*C5orf35*	1.36e-8	Estrogen receptor positive breast cancer	(Li et al., 2013) [53]
rs889312	rs331499	0.56	*C5orf35*	1.16e-11	Estrogen receptor positive breast cancer	(Li et al., 2013) [53]
rs889312	rs331499	0.56	*MIER3*	7.75e-6	Estrogen receptor positive breast cancer	(Li et al., 2013) [53]

### Nine-hundred-and-twenty-one High LD SNPs at Enhancers

**Figure 4:**
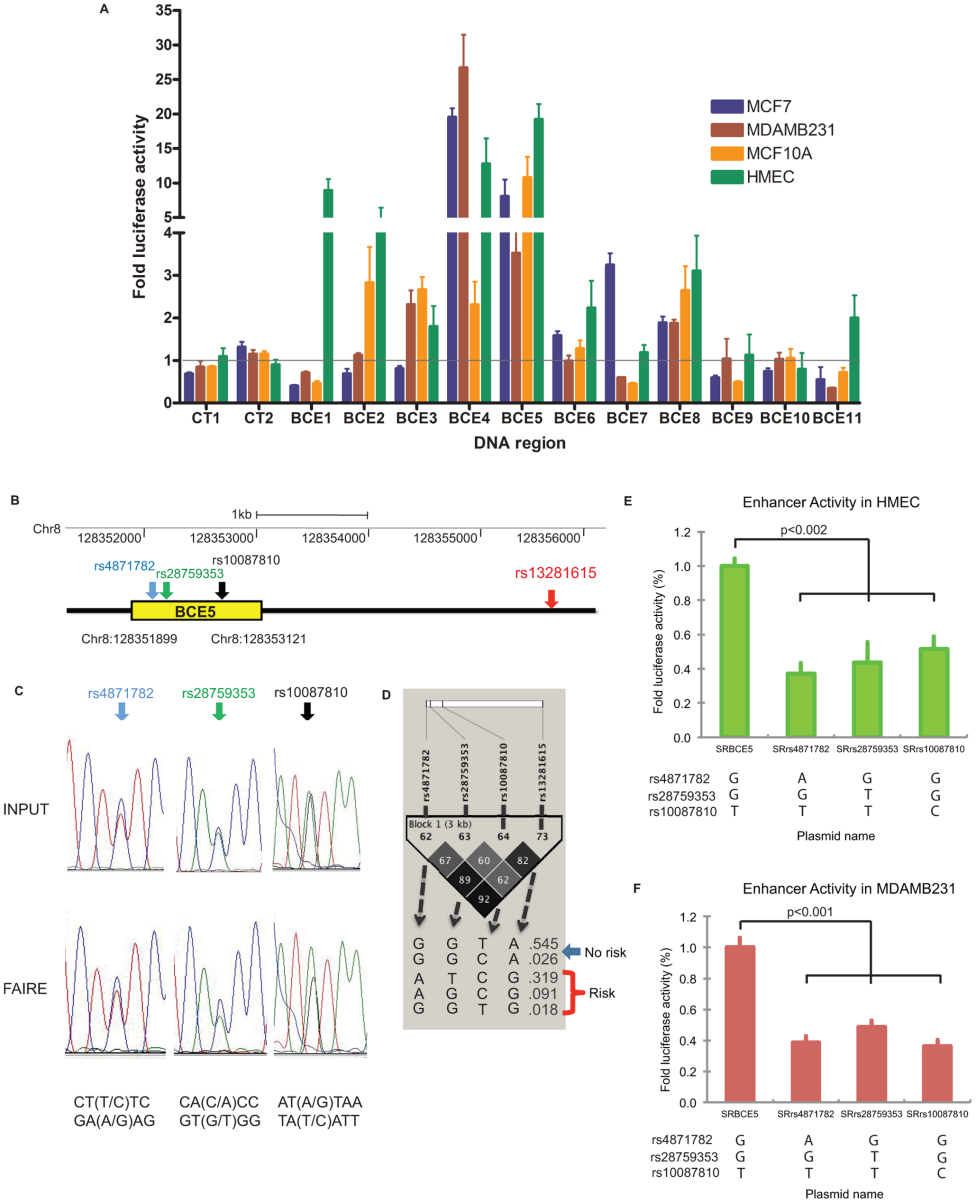
Novel enhancers including high LD SNPs were identified in breast epithelial cells. (A) Eleven enhancer regions, which included FunciSNP identified BCa high LD SNPs in epigenetically defined enhancers, were cloned and analyzed using the dual luciferase assays in MCF7 (blue), MDAMB231 (red), MCF10A (orange) and HMEC (blue). Each luciferase activity was divided by average luciferase activity of two negative controls, CT1 and 2. The average value of two negative controls was shown as a horizontal line across the breast cancer enhancers (BCEs) (gray). (B) The location of three SNPs (blue: rs4871782, green: rs28759353, black: rs10087810) at BCE5 and breast cancer risk tagSNP (red: rs13281615) in 8q24.21 region. (C) Allele-specific FAIRE assays were performed near three candidate SNPs for breast cancer risk at BCE5. Sequence results of Input DNA and FAIRE DNA are shown. The colors of the nucleotides from DNA sequencing: blue is C, green is A, black is G and red is T. Sequences near SNP were shown in a double-strand DNA (bottom). (D) Linkage Disequilibrium (LD) plot (r^2^) and haplotypes of three SNPs (in EUR) with breast cancer risk tagSNP, rs13281615 were shown [5]. Allele-specific in vitro dual luciferase assays were performed in HMEC (E) and MDAMB231 cells (F). The Analysis of variance statistical test (ANOVA) was used to confirm the difference and two-side p-values between alleles were calculated using the student t-test.

Nine-hundred-and-twenty-one high correlated SNPs (r^2^≥0.5) were annotated at enhancers (Table S9). To verify the activity of identified enhancers, we performed *in vitro* enhancer assays by cloning approximately 1.2 kb regions in which the SNPs reside. We selected the best 11 SNP regions for cloning, based on the number of chromatin biofeatures (5 or more coinciding biofeatures), and named them breast cancer enhancer 1 (BCE1) through BCE11 (Table S10). By performing dual luciferase assays in normal and breast cancer cells, we found that 9 out of the 11 regions retained enhancer activities over background (CT1 and CT2) in either normal or breast cancer cells, or in both cells types (Fig. 4A, Table S10 and S11). Among 9 active enhancers, BCE4, -5, and -8 had enhancer activities in both normal (HMEC and MCF10A) and breast cancer cells (MCF7 and MDAMB231). On the other hand, BCE1, -2, and -11 revealed enhancer activities only in normal HMEC. BCE7 had enhancer activity only in MCF7, estrogen receptor (ER) positive breast cancer epithelial cells. BCE3 retained enhancer activity in ER negative breast epithelial cells: MDAMB231, MCF10A, HMEC. These BCE enhancers were either in intron or intergenic region: BCE 3, -6, -9, -10 and -11 were in introns. Regardless of SNP location in genome, they retained enhancer activity. As an example of enhancer in intron region, BCE9 was located in intron 9 of *RAD51L1* gene, which showed differential expression level between breast cancer cells (MDAMB231) and normal breast epithelial cells (HMEC) (Fig. S4).

Among these enhancers, we investigated BCE5 in more detail as a proof-of-principle. FunciSNP analysis identified three-correlated risk SNPs (r^2^≥0.5) at the active regulatory element within BCE5 (rs4871782, rs28759353, and rs10087810) (Fig. 4B). In order to determine whether the alleles of these three SNPs participated in nucleosome depletion (i.e. as measured by FAIRE), we performed allele-specific FAIRE using a HMEC cell strain, which was heterozygous for the three SNPs. Allele-specific FAIRE can determine functional regulatory polymorphisms [62]. Here, allele-specific FAIRE for the three SNPs was performed by sequencing across the interested SNP region of FAIRE samples and comparing the sequence of peaks with that of input DNA (as control). For rs4871782, the FAIRE sample contained about the same relative amount of the two alleles, compared to input. In contrast, for rs28759353, the FAIRE sample had clearly more of the G allele, compared to the input signal. Similarly, for rs10087810, more of the T allele was detected in the FAIRE sample, compared to the input (Fig. 4C). Note the high fidelity of the sequence reactions between the FAIRE and input samples as reflected by the almost identical relative sizes of the peaks surrounding the SNP. These results may indicate that the rs28759353, G allele and rs10087810, T allele (i.e. the GT haplotype) had a more open chromatin structure than the other alleles and perhaps consequently a higher enhancer activity, which we tested next (see below).

We analyzed the haplotype of rs28759353, rs4871782, and rs10087810 SNPs relative to the risk tagSNP, rs13281615 [5]. The GGTA haplotype (Fig. 4D) had lower risk of breast cancer because it correlated with the risk allele of rs13281615. The other haplotypes and relative percentages are shown in Europeans. In order to relate allele-specific FAIRE results to enhancer activity, we next performed allele-specific *in vitro* enhancer assays by generating plasmids, which contain different versions of each SNP in BCE5 region (Fig. 4E and F). Overall, we found that the risk versions of each SNP independently had lower enhancer activities. These results together with the allele specific FAIRE data indicated that rs28759353 and rs10087810 were functional SNPs, with the risk allele having more nucleosome depletion and higher enhancer activity in the *in vitro* assay. Although we do not understand the disparity between the two assays for SNP rs4871782, it is probably related to the sensitivity of the two assays. For this particular SNP, allele-specific FAIRE is less sensitive to be picked up in the allele-specific FAIRE analysis.

### Transcription Factors, which Likely Bind to High LD SNPs at Enhancers

Among 921 SNPs in enhancer regions, 503 SNPs likely affect known transcription factor binding by altering their REs (Table S12). By performing *in silico* searches of TF REs as described above for TSS regions, we identified 455 different transcription factor REs where the TF binding will likely to be altered by the risk-correlated SNP. Among the motifs, we ranked them by the number of SNPs affecting their RE. The top 18 motifs were selected for further analysis (see below) (Table S13). The top motif was for the T-cell acute lymphocytic leukemia 1 (TAL1; aka SCL); 28 enhancer SNPs at 16 BCa risk loci were thus identified. The next ranked motifs most often likely affected in this manner were in order, Eomesoderim (EOMES), Foxhead box P1 (FOXP1) and SP1. TAL1 is a transcription factor that acts in hemopoiesis, anti-apoptosis, angiogenesis, and other activities [63], [64], [65]. It is expressed in breast tissue and decreases 2–3 fold during invasive breast carcinogenesis [42], [45] (Fig. S5). It also inhibits the expression level of GATA3, a transcription factor, which inhibits breast cancer metastasis [66], [67].

**Figure 5:**
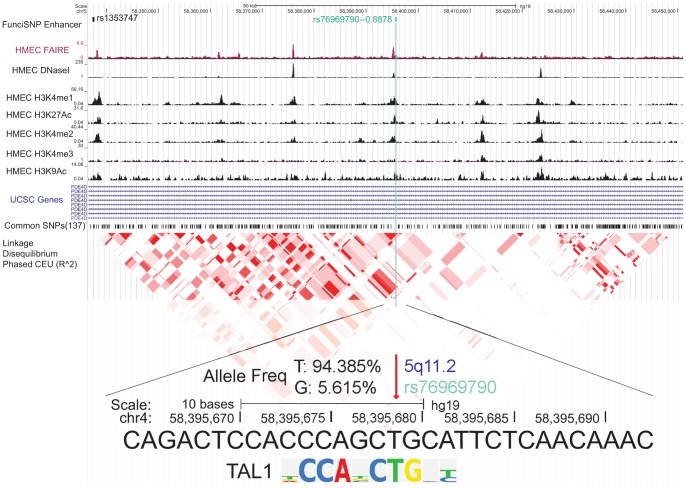
An example of enhancer SNPs, rs76969790 likely alters a TAL1 response element. The genomic browser view was shown of an enhancer SNP, rs2303696. First track showed FunciSNP results for enhancers. The name of correlated SNP (rsnumber – r^2^ value) was shown and color-coded to indicate the number of biofeatures (Fig. S3B). The index SNP was shown in black. The bottom tracks were biofeature tracks, RefSeq genes/mRNA/Pseudogene tracks from UCSC Genes, common SNPs (version 137), and Linkage Disequilibrium (LD) blocks. LD block, which was measured by r^2^ value in phased CEU is shown. Allele frequencies of rs76969790 (in all populations) and the location of this SNP in TAL1 RE are shown.

One example of a likely TAL1-affecting SNP is rs76969790 at the 5q11 risk locus (Fig. 5, Fig. S3B). The SNP is highly correlated (r^2^ = 0.88) with a GWAS index SNP, rs1353747, which is located 58 kb upstream from it. This correlated SNP is located in the large intron 10 of the *PDE4D* gene. *PDE4D* encodes for an enzyme that has 3′, 5′-cyclic-AMP phosphodiesterase activity and degrades cAMP, resulting in regulation of multiple signaling pathways and metabolism (i.e. GPCR and TOR signaling, cAMP metabolism) [68], [69]. The intron 10 of *PDE4D* gene is large, 140 kb in length and contains several histone marks of enhancers with nucleosome depletion signals (i.e. DNaseI and FAIRE). The rs76969790 is in close proximity with FAIRE and DNase1 signals and coincides exactly with enhancer histone marks (H3K4me1, H3K27ac, H3K4me2, and H3K9ac) (Fig. 5).

Additionally, expression quantitative trait locus (eQTL) analyses on the 921 high LD SNPs in enhancers were conducted using published data as we described above for TSS regions [53], [54], [55], [56], [57], [58], [59], [60], [61]. Since the eQTL analyses were detecting relationships between SNP and nearby genes (cis-eQTL), a relatively small number of enhancer high LD SNPs (65 SNPs) were associated with mRNA levels (Table S14). This is unlike the eQTL results in TSS regional high LD SNPs referred to above. Alternatively, the sample number for eQTL analyses could have been too low to detect the association signal between risk loci and affected genes.

### Interactions among Breast Cancer Risk Loci

**Figure 6:**
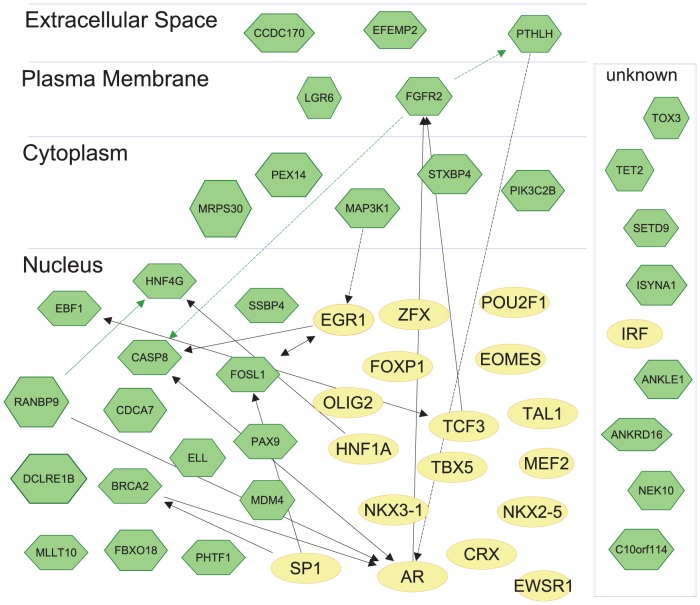
Interactions among breast cancer risk loci. 32 proteins coded by genes, which contained functional SNPs either in their exons or within their TSS regions plus the *BRCA2*, in which a nonsense index SNP resided, were laid out using subcellular localization annotation. Each molecule was shown in green hexagon. Interactions among these 33 genes/proteins were shown in green arrows (direct: solid line, indirect: dashed line). Each circle colored in yellow represents each TF. The interactions between the group, containing 33 genes/proteins and another group, containing top 18 TFs (yellow circle) that affected by high LD SNPs were shown in black arrows (direct: solid line, indirect: dashed line).

In order to investigate the interactions among genes at the breast cancer risk loci, we further highlighted 32 genes, which contained functional SNPs either in their exons or within their TSS regions. Using these 32 genes plus the *BRCA2* gene, in which rs11571833, a nonsense index SNP resided, we executed an ingenuity pathway analysis (IPA, www.ingenuity.com). When we examined interactions among these genes and/or their protein products by using data from published papers, we found only one direct interaction and two indirect interactions [70], [71], [72] (Fig. 6).

We next analyzed the relationships among the top 18 TF motifs affected by 10 or more enhancer SNPs and proteins encoded by the above 33 genes. Although some of genes have been understudied and currently lack information about their functions and locations, we observed that a number of proteins interacted with each other, and these TFs mediated interactions among the 33 BCa risk genes/proteins (Fig. 6). For instance, SP1 binds to the promoter (−329bp to 324bp) of the *FOSL1* gene, whereas SP1 binds directly to another BCa risk protein, BRCA2 [73], [74]. BRCA2 binds to several fragments of the AR protein (1aa-556aa, 627aa-919aa) [75]. In turn, AR binds to RANBP9 and CASP8 [76], [77], [78]. In prostate cancer cell lines, RANBP9 increases activity of AR protein. CASP8 protein level increases cleavage of AR proteins [76], [77], [79], [80]. These functional networks among identified genes/proteins and motifs at the 71 BCa risk loci may be key interactions, which affect genetic risk for BCa.

Recently, Cowper-Sal Lari et al [23] reported that FOXA1 binding to high LD SNPs in BCa are more frequent than other transcription factors. However, that study was performed using a limited number of transcription factors (ChIP-seq data in ER positive breast cancer cells of only 16 transcription factors) and a relatively small number of SNPs (obtained from the limited Hapmap datasets) in only 44 BCa risk loci. For an updated, more comprehensive and unbiased analysis, we assessed high LD SNPs in TF REs within TSS regions and enhancers using the 1000 genomes database, which contained not only rare variants but also un-tagged SNPs from the Hapmap project [27], [28], [81]. We further interrogated thousands of TF motifs in known datasets [47], [48], [49], [50]. Our top potentially affected TFs were SP1 and TAL1, at TSS regions and enhancers, respectively. SNPs in FOXA1 REs were ranked only 51t^h^ in our priority list (Table S12). The difference between the Cowper-Sal Lari et al [23] study and the work reported here, is likely due to our more comprehensive analysis coupled with the limited number of TFs and SNPs assessed in the Cowper-Sal Lari et al study.

Recently, it was reported that the average number of distal elements interacting with a TSS was 3.9, and the average number of TSSs interacting with a distal element was 2.5 [18]. Another study on genome structure also revealed that active chromatin regions formed inter-chromosomal contacts and blocks of each chromosome interacted with blocks in different chromosomes, composing a spatial nuclear structure [82]. Therefore, a large number of chromosomal contacts and interactions likely are orchestrated by the three-dimensional organization of the nucleus. Through eQTL analyses, we identified the precise genomic loci (SNPs) that regulated expression level of mRNAs. However, it did not demonstrate direct interactions among regulatory elements. Looping interactions between enhancers and target genes can be detected by 3C (chromatin chromosome capture) assays [18]. To scan the interactions genome-wide, 3C derivative methods (3C-seq, 4C-seq, 5C-seq, ChIA-PET and HiC-seq) may be applied [83], [84]. Targets of regulatory elements can be also identified *in vitro* and *in vivo* by knock-out DNA method such as transcription activator-like effector nucleases (TALEN) [85] and transgenic mouse modeling by knocking in conserved regulatory elements [86].

Newly identified regulatory elements, coinciding with high LD SNPs are not necessarily targeting protein-coding genes. For instance, they can interact with long noncoding RNAs (lncRNA) [87]. Each SNP identified by FunciSNP [29] was further annotated by us for proximity to the nearest known lncRNA (Table S4 and S9). We also identified potentially functional high LD SNPs in regulatory elements that intersect with lncRNA using LNCpedia database version 1.2 [88] (Table S15).

### Conclusions

Since 2005, over 1,600 variants have been identified at p-value ≤5×10^−8^ for over 250 traits. Most of the identified index SNPs from GWASs are in noncoding DNA regions, making the assignment of functionality difficult [27]. Despite the controversy surrounding the utility of GWAS, post-GWAS identification of mechanisms have become valuable for the identification of genomic targets of diseases. Here, we provide functional rationales for 21 SNPs in exons, 76 SNPs in TSS regions and 921 SNPs in putative enhancers at 60 of the 71 BCa risk loci. These annotations are based on the assumption that functional alleles are common. This short list out of more than 320,000 correlated risk SNPs can be used in follow-up fine-mapping and functional studies on identifying disease-causing SNPs.

## Materials and Methods

### Cell Culture

HMEC cells were obtained from Lonza (Lonza, Walkersville, MD) and cultured under recommended conditions. MDAMB231, MCF10A and MCF7 cells were obtained from American Type Culture Collection (ATCC, Manassas, VA). MDAMB231 and MCF7 cells were cultured in DMEM with 5% FBS. MCF10A cells were cultured in DMEM/F12 with 5% horse serum, 100 units/ml penicillin, 0.1 mg/ml streptomycin, 0.5 µg/ml hydrocortisone, 100 ng/ml cholera toxin, 10 µg/ml insulin, and 20 ng/ml epidermal growth factor (EGF).

### FAIRE-seq Library Construction and Sequencing

FAIRE assays were performed as described [89], with a number of modifications. Briefly, the method was as follows: (1) intact cells were crosslinked (1% formaldehyde in PBS); (2) nuclei were extracted from cells and re-suspended in SDS lysis buffer; (3) chromatin DNA was fragmented by sonication; (4) FAIRE DNA samples and reverse-crosslinked input DNA were purified by phenol-chloroform extraction. Two independent libraries were made for each sample by using bar-coded adapters. Each library was PCR amplified and confirmed by quantitative real-time PCR (qPCR). Single-end DNA sequencing (Illumina Hi-Seq 50 cycles) was performed at the USC Epigenome Center. Two independent assays were analyzed separately and then the data were combined in order to increase the depth of coverage (Table S16 and Fig. S6). More than 82% of the merged FAIRE peaks intersected. FAIRE-seq data were deposited in the NCBI GEO under accession number GSE46074.

### Identification of FAIRE-seq Peaks

Each bam file was filtered using a quality filter score of 30 after removing PCR artifacts and duplicates by the Samtools [90]. The identification of FAIRE-seq peaks was performed using the findPeaks from HOMER (http://biowhat.ucsd.edu/homer) [47]. Peaks were identified by using a triangle-based distribution with a median length of 150bp. In order to find the peaks, which are not false positive, we used input with an alpha value of 0.01; 99.0% confidence interval for peak pairs, which are unequal between sample and input was used. A subpeak value of 0.6 with a trim float value of 0.3 was used to perform peak separation. After peak identification, we calculated a p-value for each peak between sample and input. To be most stringent, functional peaks [47] at a p-value of 10^−9^ were used as a cut off to select significantly enriched peaks. FAIRE-seq data within 3 kb windows centered on the annotated TSS of genes were used to define TSS regions. The data >1.5 kb from TSS were utilized to define enhancer regions for the FunciSNP analysis.

### Histone Modification ChIP-seq Data

Histone modification ChIP-seq data (H3K4me1, me2, me3, H3K9Ac and H3K27Ac) in HMEC were obtained from accession number [GSE29611] through the NCBI Gene Expression Omnibus portal. [GSE29611] was published as part of the ENCODE project. ChIP assay protocol as well as data processing details may be seen here (http://genome.ucsc.edu/cgi-bin/hgTrackUi?db=hg19&g=wgEncodeBroadHistone).

Chromatin State Segmentataion HMM data generated by using above ChIP-seq data were obtained from accession number [GSE38163] and included for the FunciSNP analyses of regulatory elements. NGS data within 3 kb windows centered on the annotated transcription start sites of genes were used for TSS regions. For putative enhancer regions, NGS data >1.5 kb from TSS were utilized.

### DNaseI-seq Data

DNaseI-seq data in HMEC were obtained from accession number [GSE32970] through the NCBI Gene Expression Omnibus portal. Additional DNaseI-seq data generated by University of Washington as part of the ENCODE project were downloaded from here (http://hgdownload.cse.ucsc.edu/goldenPath/hg19/encodeDCC/wgEncodeUwDnase/). Detailed protocols may be seen at following websites (http://genome.ucsc.edu/cgi-bin/hgTrackUi?hgsid=307403817&c=chr1&g=wgEncodeOpenChromDnase and http://genome.ucsc.edu/cgi-bin/hgTrackUi?hgsid=307403817&c=chr1&g=wgEncodeUwDnase). NGS data within 3 kb windows centered on the annotated transcription start sites of genes were used to define TSS regions for FunciSNP analysis. For putative enhancer regions, NGS data >1.5 kb from TSS were utilized.

### FunciSNP

FunciSNP is an in-house developed R/Bioconductor package for the Functional Integration of SNPs with phenotype by coincidence with chromatin biofeatures. All statistical tests were done using R software (R version 2.9.2, 2009-08-24, (R Development Core Team, 2009)). FunciSNP version 0.99 was used to find correlated SNPs, which coincide with 11 independent ChIP-seq/FAIRE-seq/DNaseI-seq data sets in TSS regions and putative enhancer regions. All the SNPs from the 1000 genomes project (up to May 2012 data release) [28] residing in 1 Mb windows around breast cancer risk index SNP and within EUR ethnic groups (original GWAS), were analyzed with an r^2^ value 0.5 as a cut-off (Table S4 and S9).

### Plasmid Construction and Luciferase Reporter Assays

Eleven potential enhancer regions (∼1200bp sequence surrounding the nucleosome depleted regions with FunciSNP identified correlated SNP) were amplified from genomic DNA using High Fidelity Platinum Tag DNA polymerase (Invitrogen Corp., Carlsbad, CA). The amplified sequences were then subcloned using SacII, EcoRI, BglII or KpnI restriction sites upstream of a thymidine kinase (TK) minimal promoter-firefly-luciferase vector. All clones were confirmed by sequencing. The primer sequences for subcloning are listed in Table S11. HMEC, MCF10A, MDAMB231, MCF7 cells were transfected with reporter plasmids along with constitutively active pRL-TK Renilla luciferase plasmid (Promega Corp., Madison, WI) using Lipofectamine LTX Reagent (Invitrogen Corp., Carlsbad, CA) under recommended protocol. Dual luciferase activities were measured as previously described [91].

### Allele-specific FAIRE

PCR reactions were performed on FAIRE-isolated and input DNA using High Fidelity Platinum Taq DNA polymerase (Invitrogen Corp., Carlsbad, CA) for 15 cycles after which products were purified and re-PCRed for 20 cycles to minimize the PCR artifacts due to over-cycling. Purified DNA from these reactions was sequenced, using primers near the SNP locations by the DNA Core Facility at the University of Southern California (Table S11). Each experiment was independently performed more than twice.

### Allele-specific Luciferase Reporter Assays

Point mutations were introduced to create enhancer-reporter constructs with specific SNP allele using QuikChange site-directed mutagenesis kit (Agilent Technologies Inc., Santa Clara, CA). In order to avoid the bias from miniprep procedures, six independent clones of each construct were made and confirmed by sequencing. Each of the six independent clones of each construct were transfected in four wells and two luciferase assays per well were performed in order to record luciferase-reading variation. Allele-specific fold activities were presented and values shown are means ± SEM of the six independent clones of each allele. The analysis of variance statistical test (ANOVA) was used to confirm the difference and two-side p-values between alleles were calculated using the student t-test.

### Gene Expression Analysis between Breast Cancer and Normal Breast Tissues

We compared gene expression levels between breast cancer and normal tissues using the Oncomine database, released in Sep 2012 [45]. This database currently contained more than 674 datasets and information on 73,327 samples tissues, including datasets with over 593 samples for breast cancer [39], [40], [41], [42], [43], [44], [45]. For the differential expression analyses, t-test with false discovery rates as a corrected measure of significance was performed and following cut-off thresholds were utilized: p-value <10^−4^, fold change >2.0, within top 10% gene rank. The result of this analysis for the genes, which high LD TSS regional SNPs reside in, is listed in Table S5. As an example, *TAL1* gene expression level change between normal and breast cancer tissues were shown in detail as boxplots (Fig. S5).

### RNA-seq Data for the *ANKLE1* Gene

Long RNA-seq from ENCODE/Cold Spring Harbor Lab in HMEC and MCF7 cells were obtained through the UCSC genome browser tracks [92]. In addition to profiling Poly-A+ and Poly-A- RNA from whole cells, RNA-seq data from the cytosol and nucleus were performed in MCF7 cells. These expression data at the *ANKLE1* gene were shown in Fig. S2.

### Gene Expression Analysis between HMEC and MDAMB231 Cells

We compared gene expression levels between HMEC and MDAMB231 cells by using the affymetrix HG-U133 plus2 microarrays obtained from the accession number [GSE33167] [93]. *RAD51L1* gene expression values for both cells were processed and its bar plots were graphed by using the GEO2R [94] (Fig. S4).

### eQTL Analyses

We performed expression quantitative trait locus (eQTL) analyses on FunciSNP identified SNPs to examine whether these SNPs were associated with messenger RNA (mRNA) level of nearby genes. We assessed eQTL for all SNPs by using the RegulomeDB, the GTEX database (http://www.ncbi.nlm.nih.gov/gtex/GTEX2/gtex.cgi), University of Chicago eQTL Browser (http://eqtl.uchicago.edu), the Genevar (http://www.sanger.ac.uk/resources/software/genevar/), and The Cancer Genome Atlas (TCGA) breast cancer datasets in 15 breast cancer risk loci [53], [54], [55], [56], [57], [58], [59], [60], [61]. To be most stringent, a p-value of 10^−5^ was used as a cut-off (Table S8 and S14). Posterior probability and the Bayes factor were used to analyze the eQTL data from Veyrieras et al and Mangravite et al [59].

### Motif Discovery

In order to annotate SNP effects on regulatory motifs, sets of position weight matrices (PWMs) were used from FIMO, HOMER (ChIP-seq known motifs), Genome Trax (ChIP-seq TFBS), Haploreg (TRANSFAC, JASPAR, and PBM) [47], [48], [49], [50]. FIMO analysis was performed using the motif database, called JASPAR CORE 2009 vertebrates, downloaded from the MEME suite (http://tools.genouest.org/tools/meme/meme-download.html) [48]. P-value for output threshold utilized for FIMO was 1e-4. FindMotif analysis was executed by using known motifs generated from HOMER. Each motif matrix was established after collecting strong binding sites of each TF genome wide from published human ChIP-seq data. Log odds score of the motif matrix cut-off value 5 was used for findMotif analysis. Predicted ChIP-seq TFBS analysis from Genome Trax was utilized with the motif score cut-off 0.7. Its database contains motif matrices from best-scoring TF binding sites identified with a ChIP-chip or ChIP-seq fragment. A stringent threshold of p<4^−8^ was applied for the PWM score of each instance for Haploreg. The change in log-odds (LOD) score as alleles change was calculated and listed in Table S6 and S9. Each identified motif RE was organized by SNP id, and the number of SNPs affecting regulatory motif was counted to rank the TFs (Table S6 and S12).

### Transcription Factor and Gene/protein Interaction Analysis

We obtained information of the top 18 TFs and 33 genes/proteins using an Ingenuity Pathway Analysis (IPA, www.ingenuity.com). IPA Path Explore tools were used to identify direct and indirect interactions among molecules. IPA Path Designer tools were utilized to map the annotated subcellular location of each molecule.

## Supporting Information

Figure S1 **Linkage Disequilibrium block and haplotype analysis of 2 corrSNPs, rs8100241 and rs8108174, and their index SNP, rs2363956.** (A) Linkage Disequilibrium block (in EUR) showing two high LD SNPs, rs8100241 and rs8108174, and index SNP, rs2363956, found in exons of *ANKLE1*. (B) Haplotypes of these SNPs (in EUR) and protein isoforms, containing different amino acid compositions. Antoniou et al reported that T allele of rs2363956 is associated with breast cancer risk [4]
(TIF)

Figure S2 **The UCSC genome browser near the** ***ANKLE1*** **gene, showing breast epithelial cell RNA-seq data.** Long RNA-seq from ENCODE/Cold Spring Harbor Lab in HMEC and MCF7 cells were used [92]. For MCF7 cells, in addition to profiling Poly-A+ and Poly-A- RNA from whole cells, RNA-seq data from the cytosol and nucleus were performed. Two replicates for each condition were conducted. Contigs and signals from each replicate were shown in the above tracks.
(TIF)

Figure S3 **Overlap count keys for FunciSNP results. The name of correlated SNP is colored based on the number of biofeatures.** (A) Overlap count key for FunciSNP results for TSS regions. (B) Overlap count key for FunciSNP results for enhancers.
(TIF)

Figure S4 ***RAD51L1*** **gene expression value in HMEC and MDAMB231.** *RAD51L1* gene expression value for HMEC and MDAMB231 were obtained from accession number [GSE33167]. Three replicates for each cell type were generated by using the affymetrix HG-U133 plus2 arrays [93]. Expression bar plots were graphed by using the GEO2R [94]
(TIF)

Figure S5 ***TAL1*** **expression level in breast tissues.** The expression value of *TAL1* gene was obtained from The Cancer Genome Atlas (TCGA) breast tissues [42]. (A) *TAL1* expression level comparison between normal breast tissues and invasive breast carcinoma (B) comparison between normal breast tissues and invasive ductal breast carcinoma (C) comparison between normal breast tissues and mixed lobular and ductal breast carcinoma (D) comparison between normal breast tissues and invasive lobular breast carcinoma. The analysis was performed by using the Oncomine database [95].
(TIF)

Figure S6 **HMEC FAIRE peaks from two replicates.**
(TIF)

Table S1 **71 Breast cancer risk index SNPs and high LD SNPs genomic locations.**
(DOC)

Table S2 **Protein function prediction results for missense variants of high LD SNPs.**
(XLS)

Table S3 **Protein function prediction of index SNPs in exons.**
(XLS)

Table S4 **FunciSNP results for TSS regional high LD SNPs.**
(XLS)

Table S5 **Differential expression analysis of the genes, which high LD TSS regional SNPs reside in.**
(XLS)

Table S6 **TSS regional high LD SNP motif analysis result.**
(XLS)

Table S7 **Top 10 TF motifs for TSS regional high LD SNPs.**
(DOC)

Table S8 **eQTL analyses on 76 TSS regional high LD SNPs.**
(DOC)

Table S9 **FunciSNP results for high LD SNPs in enhancers.**
(XLS)

Table S10 **Breast Cancer Enhancer (BCE) regions used for luciferase assays.**
(DOC)

Table S11 **Oligonucleotide sequences used for cloning and qPCR.**
(DOC)

Table S12 **high LD SNPs in enhancer motif analysis result.**
(XLS)

Table S13 **Top 18 TF motifs for high LD SNPs in enhancers.**
(DOC)

Table S14 **eQTL analyses on high LD SNPs in enhancers.**
(DOC)

Table S15 **lncRNA which intersect with high LD SNPs in regulatory elements.**
(XLS)

Table S16 **FAIRE-seq statistics.**
(XLS)
