# Supplementary figures and images for: Comprehensive Functional Annotation of Seventy-One Breast Cancer Risk Loci
Source: PLoS One. 2013 May 22;8(5):e63925. doi: 10.1371/journal.pone.0063925 (PMC3661550; doi:10.1371/journal.pone.0063925)

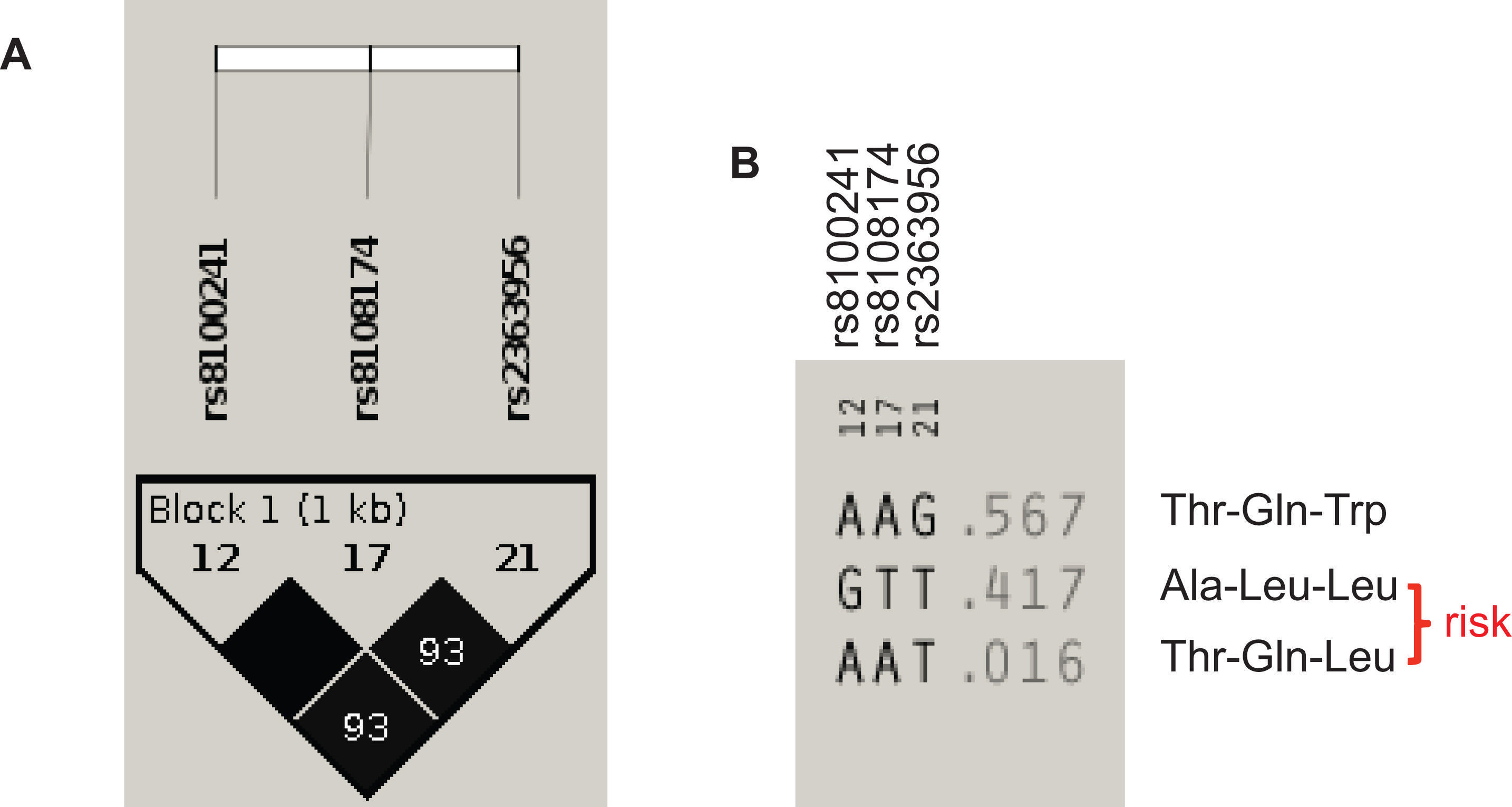

Supplement: Figure S1 — Linkage Disequilibrium block and haplotype analysis of 2 corrSNPs, rs8100241 and rs8108174, and their index SNP, rs2363956. (A) Linkage Disequilibrium block (in EUR) showing two high LD SNPs, rs8100241 and rs8108174, and index SNP, rs2363956, found in exons of ANKLE1. (B) Haplotypes of these SNPs (in EUR) and protein isoforms, containing different amino acid compositions. Antoniou et al reported that T allele of rs2363956 is associated with breast cancer risk [4] (TIF) [file pone.0063925.s001.tif]

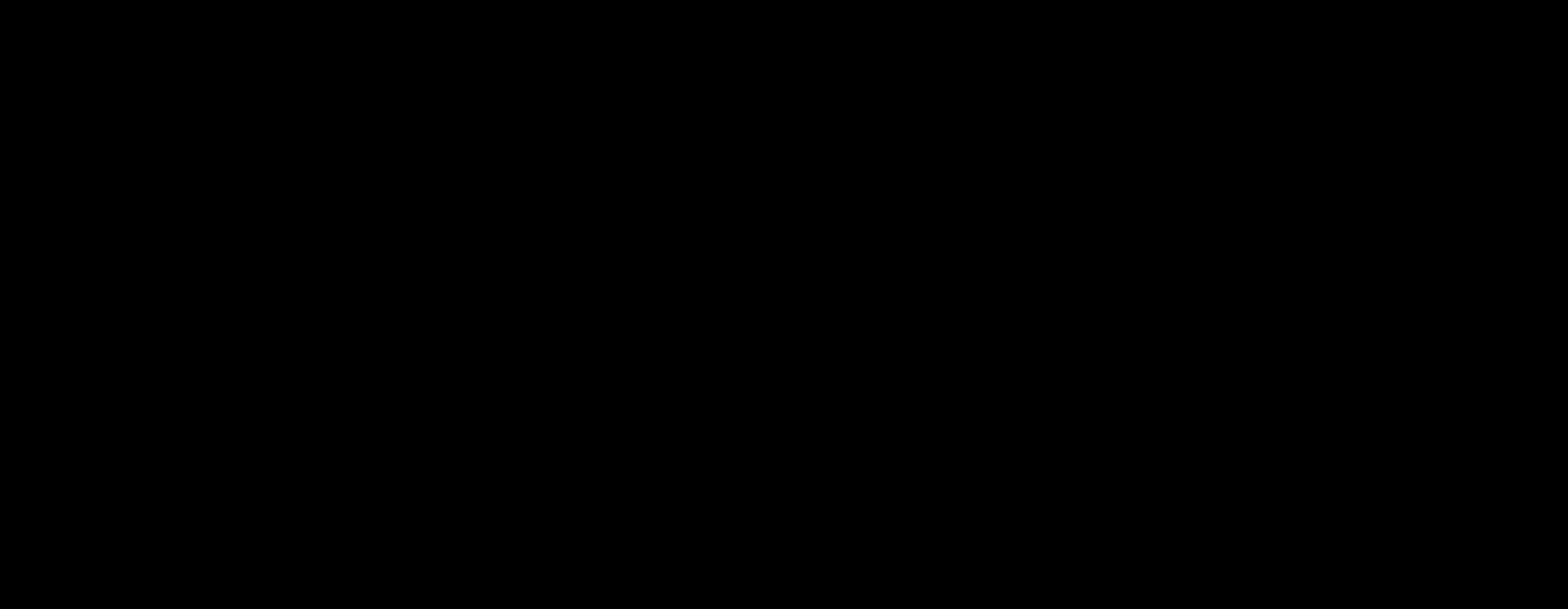

Supplement: Figure S2 — The UCSC genome browser near the ANKLE1 gene, showing breast epithelial cell RNA-seq data. Long RNA-seq from ENCODE/Cold Spring Harbor Lab in HMEC and MCF7 cells were used [92]. For MCF7 cells, in addition to profiling Poly-A+ and Poly-A- RNA from whole cells, RNA-seq data from the cytosol and nucleus were performed. Two replicates for each condition were conducted. Contigs and signals from each replicate were shown in the above tracks. (TIF) [file pone.0063925.s002.tif]

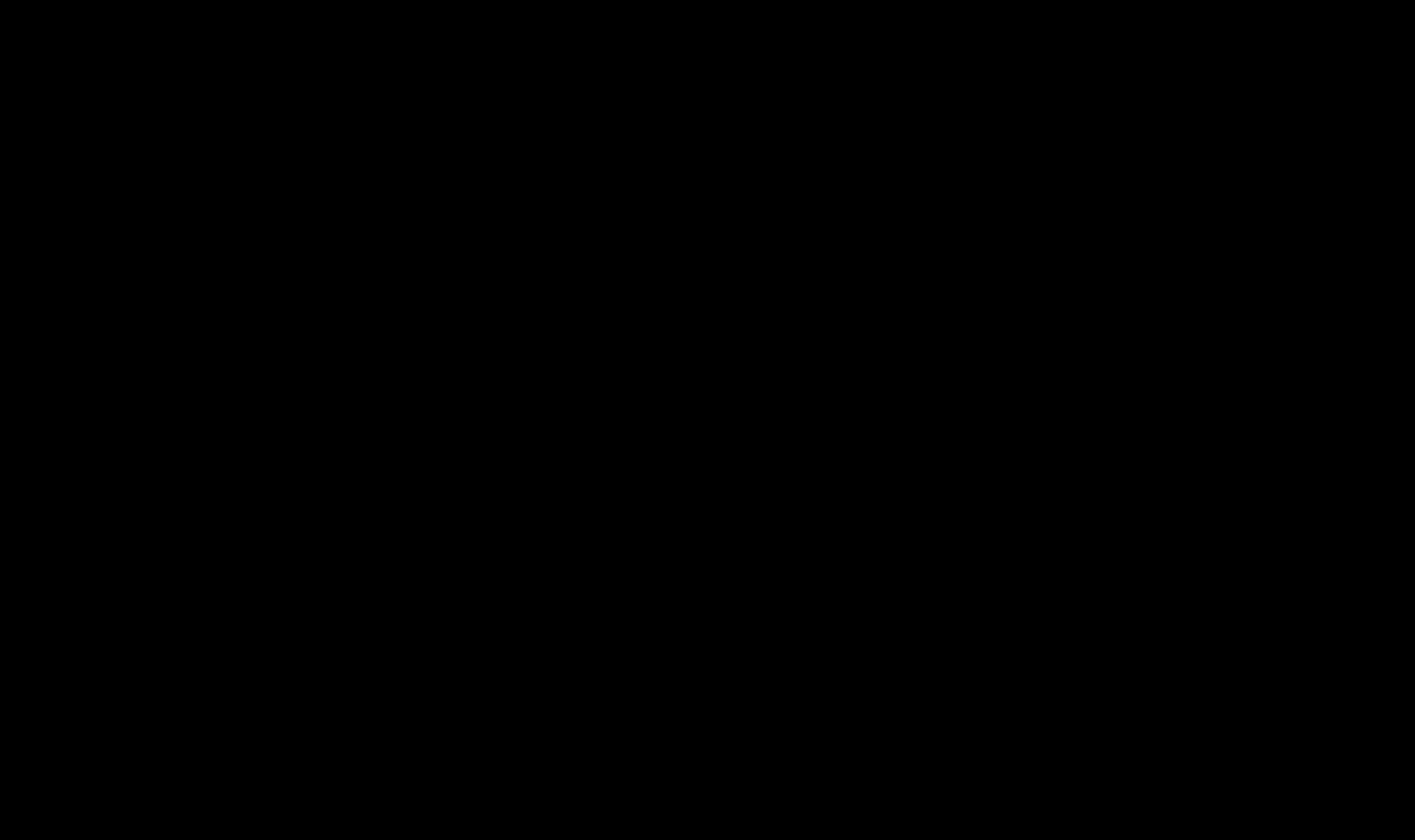

Supplement: Figure S3 — Overlap count keys for FunciSNP results. The name of correlated SNP is colored based on the number of biofeatures. (A) Overlap count key for FunciSNP results for TSS regions. (B) Overlap count key for FunciSNP results for enhancers. (TIF) [file pone.0063925.s003.tif]

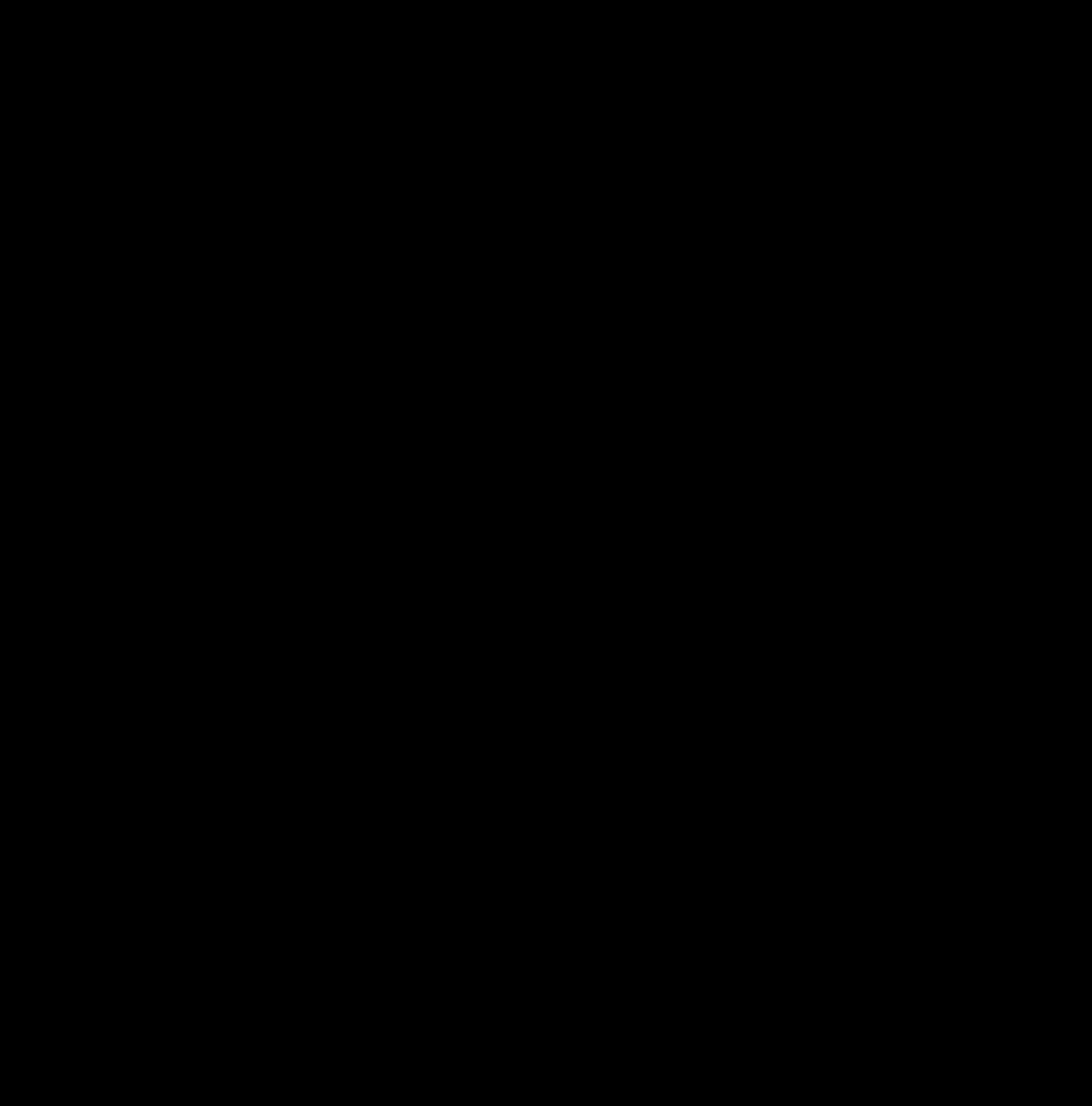

Supplement: Figure S4 — RAD51L1 gene expression value in HMEC and MDAMB231. RAD51L1 gene expression value for HMEC and MDAMB231 were obtained from accession number [GSE33167]. Three replicates for each cell type were generated by using the affymetrix HG-U133 plus2 arrays [93]. Expression bar plots were graphed by using the GEO2R [94] (TIF) [file pone.0063925.s004.tif]

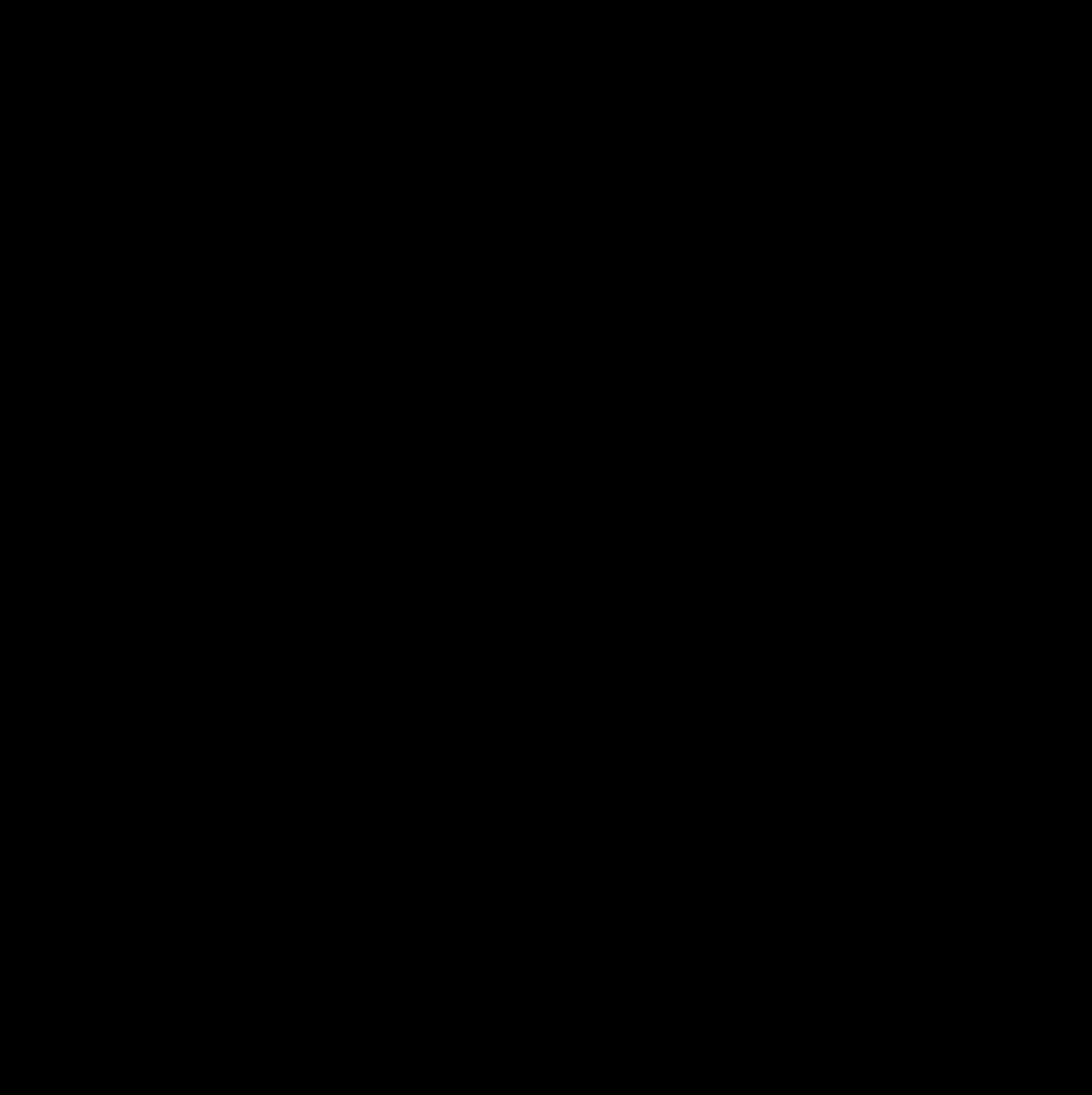

Supplement: Figure S5 — TAL1 expression level in breast tissues. The expression value of TAL1 gene was obtained from The Cancer Genome Atlas (TCGA) breast tissues [42]. (A) TAL1 expression level comparison between normal breast tissues and invasive breast carcinoma (B) comparison between normal breast tissues and invasive ductal breast carcinoma (C) comparison between normal breast tissues and mixed lobular and ductal breast carcinoma (D) comparison between normal breast tissues and invasive lobular breast carcinoma. The analysis was performed by using the Oncomine database [95]. (TIF) [file pone.0063925.s005.tif]

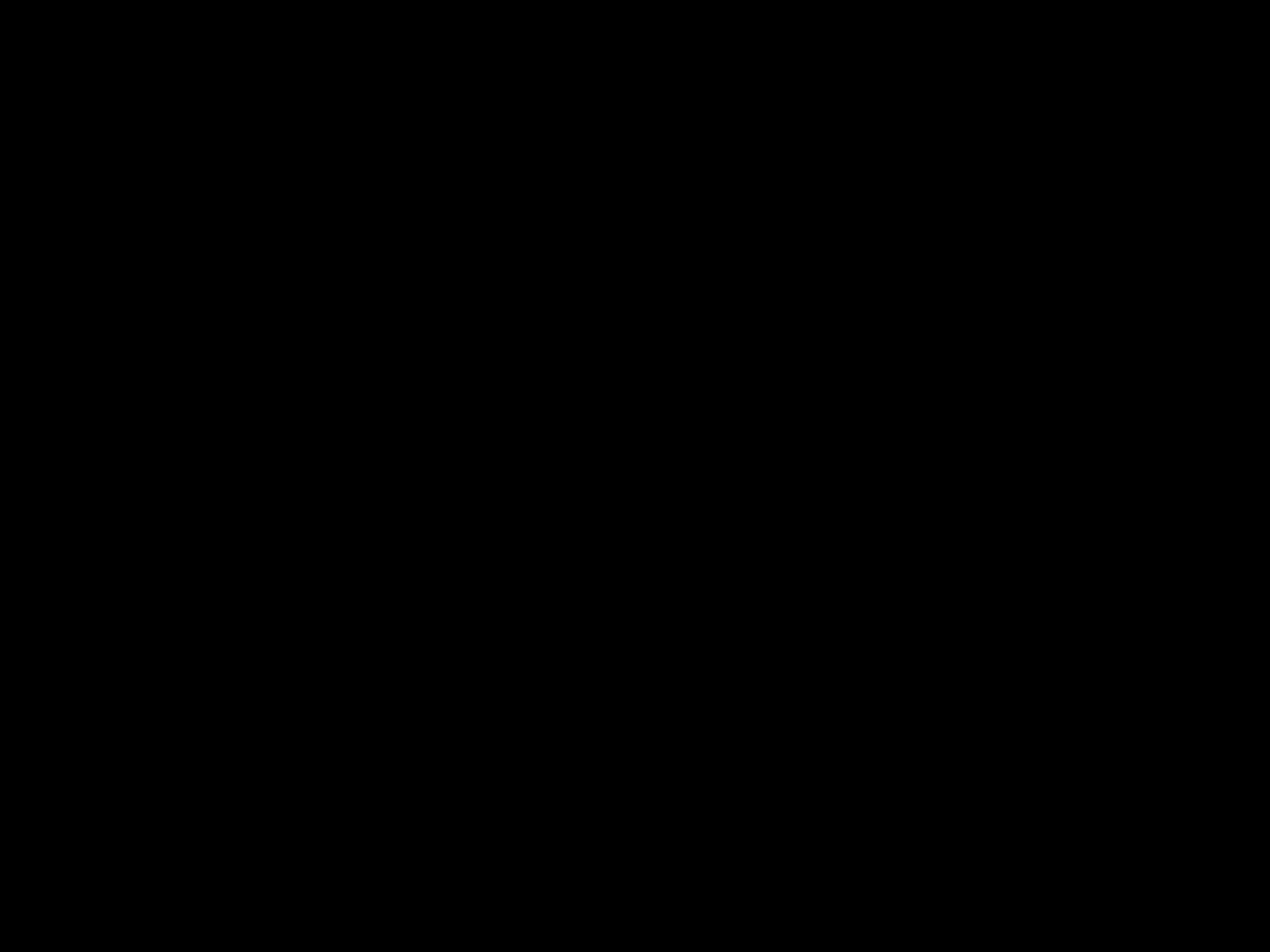

Supplement: Figure S6 — HMEC FAIRE peaks from two replicates. (TIF) [file pone.0063925.s006.tif]
