# Supplementary material for: Comprehensive Functional Annotation of Seventy-One Breast Cancer Risk Loci
Source: PLoS One. 2013 May 22;8(5):e63925. doi: 10.1371/journal.pone.0063925 (PMC3661550; doi:10.1371/journal.pone.0063925)
Supplement: Table S1 — 71 Breast cancer risk index SNPs and high LD SNPs genomic locations. (DOC) [file pone.0063925.s007.doc]

Table S1. 71 Breast cancer risk index SNPs and high LD SNPs genomic locations

| risk region # | Chr. | risk index SNP | Number of high LD SNPs within TSS region biofeature | Number of high LD SNPs within Enhancer biofeature | Number of high LD SNPs within coding Exon biofeature | Nearest gene of index SNP | Index SNP genomic location (intron, exon, intergenic) |
| --- | --- | --- | --- | --- | --- | --- | --- |
| 1 | 1p11.2 | rs11249433 |  |  |  | EMBP1 | intron |
| 2 | 1p13.2 | rs11552449 | 3 | 6 | 1 | DCLRE1B | exon(misssense) |
| 3 | 1p36.22 | rs616488 |  | 16 | 1 | PEX14 | intron |
| 4 | 1q32.1 | rs4245739 | 8 | 21 |  | MDM4 | intron(3'UTR) |
| 5 | 1q32.1 | rs6678914 | 2 | 10 | 1 | LGR6 | intron |
| 6 | 2p24.1 | rs12710696 |  | 15 |  | OSR1(200kb) | intergenic |
| 7 | 2q14.2 | rs4849887 |  | 3 |  | INHBB(100kb) | intergenic |
| 8 | 2q31.1 | rs1550623 | 3 |  |  | CDCA7(6kb) | intergenic |
| 9 | 2q31.1 | rs2016394 |  | 3 |  | DLX2(5kb) | intergenic |
| 10 | 2q33.1 | rs1045485 |  |  | 1 | CASP8 | exon(missense) |
| 11 | 2q35 | rs13387042 |  |  |  | TNP1(200kb) | intergenic |
| 12 | 2q35 | rs16857609 |  | 24 |  | DIRC3 | intron |
| 13 | 3p24.1 | rs12493607 |  | 34 |  | TGFBR2 | intron |
| 14 | 3p24.1 | rs4973768 |  | 14 | 1 | SLC4A7 | 3'UTR |
| 15 | 3p26.1 | rs6762644 |  | 15 |  | ITPR1 | intron |
| 16 | 4q24 | rs9790517 | 1 | 2 |  | TET2 | intron |
| 17 | 4q34.1 | rs6828523 |  |  |  | ADAM29 | intron |
| 18 | 5p12 | rs4415084 | 6 | 16 | 1 | MRPS30(100kb) | intergenic |
| 19 | 5p15.33 | rs10069690 |  | 1 |  | TERT | intron |
| 20 | 5q11.2 | rs10472076 |  |  |  | RAB3C(30kb), PDE4D | intergenic |
| 21 | 5q11.2 | rs1353747 |  | 3 |  | PDE4D | intron |
| 22 | 5q11.2 | rs889312 | 17 | 8 |  | MAP3K1(60kb) | intergenic |
| 23 | 5q33.3 | rs1432679 |  |  | 1 | EBF1 | intron |
| 24 | 6p23 | rs204247 | 2 | 5 | 1 | RANBP9(10kb) | intergenic |
| 25 | 6p25.3 | rs11242675 |  | 2 |  | FOXQ1(3kb) | intergenic |
| 26 | 6q14.1 | rs17529111 |  | 1 |  | FAM46A(300kb) | intergenic |
| 27 | 6q25.1 | rs2046210 | 1 | 4 | 1 | C6orf97(5kb) | intergenic |
| 28 | 6q25.1 | rs3757318 |  |  |  | C6orf97 | intron |
| 29 | 7q35 | rs720475 |  | 3 |  | ARHGEF5 | intron |
| 30 | 8p12 | rs9693444 |  | 42 |  | C8orf75(80kb), DUSP4(250kb) | intergenic |
| 31 | 8q21.11 | rs2943559 |  | 1 | 1 | HNF4G | intron |
| 32 | 8q21.11 | rs6472903 |  |  |  | HNF4G(80kb) | intergenic |
| 33 | 8q24.21 | rs11780156 |  | 42 |  | PVT1(70kb) | intergenic |
| 34 | 8q24.21 | rs13281615 |  | 38 |  | POU5F1B(100kb) | intergenic |
| 35 | 9p21.3 | rs1011970 |  | 1 |  | CDKN2A,CDKN2B | intron |
| 36 | 9q31.2 | rs10759243 |  | 2 |  | KLF4(50kb) | intergenic |
| 37 | 9q31.2 | rs865686 |  | 10 |  | KLF4(500kb) | intergenic |
| 38 | 10p12.31 | rs11814448 |  | 1 |  | DNAJC1(20kb) | intergenic |
| 39 | 10p12.31 | rs7072776 | 11 | 11 | 1 | MLLT10(300bp) | intergenic |
| 40 | 10p15.1 | rs2380205 | 2 | 11 |  | ANKRD16(25kb) | intergenic |
| 41 | 10q21.2 | rs10995190 |  |  |  | ZNF365 | intron |
| 42 | 10q22.3 | rs704010 |  | 24 |  | ZMIZ1 | intron |
| 43 | 10q25.2 | rs7904519 |  | 36 |  | TCF7L2 | intron |
| 44 | 10q26.12 | rs11199914 |  | 5 |  | FGFR2(100kb) | intergenic |
| 45 | 10q26.13 | rs2981582 | 1 | 15 |  | FGFR2 | intron |
| 46 | 11p15.5 | rs3817198 |  | 1 |  | LSP1 | intron |
| 47 | 11q13.1 | rs3903072 | 2 | 11 | 3 | SNX32(15kb), OVOL1(15kb) | intergenic |
| 48 | 11q13.3 | rs614367 |  | 1 |  | CCND1(100kb) | intergenic |
| 49 | 11q24.3 | rs11820646 |  | 1 |  | BARX2(130kb) | intergenic |
| 50 | 12p11 | rs10771399 | 1 | 62 |  | PTHLH (30kb) | intergenic |
| 51 | 12p13.1 | rs12422552 |  | 9 |  | ATF7IP(80kb) | intergenic |
| 52 | 12q22 | rs17356907 |  | 2 |  | NTN4(20kb) | intergenic |
| 53 | 12q24.21 | rs1292011 |  | 5 |  | MED13L(400kb) | intergenic |
| 54 | 13q13.1 | rs11571833 |  |  |  | BRCA2 | exon(nonsense) |
| 55 | 14q13.3 | rs2236007 | 3 |  | 1 | PAX9 | intron |
| 56 | 14q24.1 | rs2588809 |  | 39 |  | RAD51B | intron |
| 57 | 14q24.1 | rs999737 |  | 28 |  | RAD51B | intron |
| 58 | 14q32.11 | rs941764 |  |  |  | CCDC88C | intron |
| 59 | 16q12.1 | rs3803662 | 1 |  |  | TOX3(5kb) | intergenic |
| 60 | 16q12.2 | rs11075995 |  | 22 |  | FTO | intron |
| 61 | 16q12.2 | rs17817449 |  | 98 |  | FTO | intron |
| 62 | 16q23.2 | rs13329835 |  |  |  | CDYL2 | intron |
| 63 | 17q23 | rs6504950 | 7 | 10 | 1 | STXBP4 | intron |
| 64 | 18q11.2 | rs1436904 |  | 2 |  | CHST9 | intron |
| 65 | 18q11.2 | rs527616 |  | 1 |  | AQP4(80kb) | intergenic |
| 66 | 19p13.11 | rs2363956 | 2 | 2 | 2 | ANKLE1 | exon(missense) |
| 67 | 19p13.11 | rs4808801 | 3 | 49 | 3 | ELL | intron |
| 68 | 19q13.31 | rs3760982 |  | 39 |  | KCNN4(1kb) | intergenic |
| 69 | 21q21.1 | rs2823093 |  | 6 |  | NRIP1(60kb) | intergenic |
| 70 | 22q12.2 | rs132390 |  |  |  | EMID1 | intron |
| 71 | 22q13.1 | rs6001930 |  | 88 |  | MKL1 | intron |
