# Supplementary material for: Comprehensive Functional Annotation of Seventy-One Breast Cancer Risk Loci
Source: PLoS One. 2013 May 22;8(5):e63925. doi: 10.1371/journal.pone.0063925 (PMC3661550; doi:10.1371/journal.pone.0063925)
Supplement: Table S7 — Top 10 TF motifs for TSS regional high LD SNPs. (DOC) [file pone.0063925.s013.doc]

Table S7. Top 10 TF motifs for TSS regional high LD SNPs

| Motif name | number of SNPs within each TF motif’s RE |
| --- | --- |
| SP1 | 6 |
| Egr1 | 5 |
| CEBPB | 2 |
| EBF1 | 2 |
| Klf4 | 2 |
| Maz | 2 |
| Myf | 2 |
| MZF1 | 2 |
| NF1 | 2 |
| TATA-Box (TBP) | 2 |
