# Supplementary material for: Comprehensive Functional Annotation of Seventy-One Breast Cancer Risk Loci
Source: PLoS One. 2013 May 22;8(5):e63925. doi: 10.1371/journal.pone.0063925 (PMC3661550; doi:10.1371/journal.pone.0063925)
Supplement: Table S8 — eQTL analyses on 76 TSS regional high LD SNPs. (DOC) [file pone.0063925.s014.doc]

Table S8. eQTL analyses on 76 TSS regional high LD SNPs

| index SNP | High LD  SNP | r^2^ | target Gene | eQTL  P-value | Cell type | reference |
| --- | --- | --- | --- | --- | --- | --- |
| rs889312 | rs252913 | 0.59 | MGC33648 | 1.32E-149 | Monocytes | (Zeller et al., 2010) |
| rs889312 | rs331499 | 0.56 | MGC33648 | 6.99E-169 | Monocytes | (Zeller et al., 2010) |
| rs6504950 | rs7222197 | 1.00 | COX11 | 3.00E-19 | Monocytes | (Zeller et al., 2010) |
| rs2046210 | rs7763637 | 0.91 | C6orf97 | 4.85E-14 | Monocytes | (Zeller et al., 2010) |
| rs889312 | rs252913 | 0.59 | C5orf35 | 2.40E-06 | Lymphoblastoid | (Stranger et al., 2012) |
| rs889312 | rs252913 | 0.59 | C5orf35 | 6.00E-06 | Lymphoblastoid | (Stranger et al., 2012) |
| rs889312 | rs252913 | 0.59 | C5orf35 | 9.10E-06 | Lymphoblastoid | (Stranger et al., 2012) |
| rs889312 | rs252913 | 0.59 | C5orf35 | 9.50E-06 | Lymphoblastoid | (Stranger et al., 2012) |
| rs889312 | rs252925 | 0.59 | C5orf35 | 2.00E-06 | Lymphoblastoid | (Stranger et al., 2012) |
| rs889312 | rs252925 | 0.59 | C5orf35 | 2.50E-06 | Lymphoblastoid | (Stranger et al., 2012) |
| rs889312 | rs252925 | 0.59 | C5orf35 | 7.10E-06 | Lymphoblastoid | (Stranger et al., 2012) |
| rs889312 | rs331499 | 0.56 | C5orf35 | 5.60E-06 | Lymphoblastoid | (Stranger et al., 2012) |
| rs889312 | rs331499 | 0.56 | C5orf35 | 9.10E-06 | Lymphoblastoid | (Stranger et al., 2012) |
| rs889312 | rs702691 | 0.60 | C5orf35 | 1.80E-06 | Lymphoblastoid | (Stranger et al., 2012) |
| rs889312 | rs702691 | 0.60 | C5orf35 | 4.90E-06 | Lymphoblastoid | (Stranger et al., 2012) |
| rs2363956 | rs8100241 | 0.94 | PDE4C | 8.60E-06 | Lymphoblastoid | (Stranger et al., 2012) |
| rs889312 | rs832540 | 0.59 | C5orf35 | 1.80E-06 | Lymphoblastoid | (Stranger et al., 2012) |
| rs889312 | rs832540 | 0.59 | C5orf35 | 2.00E-06 | Lymphoblastoid | (Stranger et al., 2012) |
| rs889312 | rs832540 | 0.59 | C5orf35 | 2.50E-06 | Lymphoblastoid | (Stranger et al., 2012) |
| rs889312 | rs832540 | 0.59 | C5orf35 | 9.10E-06 | Lymphoblastoid | (Stranger et al., 2012) |
| rs889312 | rs832552 | 0.61 | C5orf35 | 1.80E-06 | Lymphoblastoid | (Stranger et al., 2012) |
| rs889312 | rs252925 | 0.59 | C5orf35 | 2.50E-06 | Adipose | (Nica et al., 2011) |
| rs889312 | rs252925 | 0.59 | C5orf35 | 7.00E-06 | Adipose | (Nica et al., 2011) |
| rs889312 | rs252925 | 0.59 | C5orf35 | 1.40E-09 | Lymphoblastoid | (Nica et al., 2011) |
| rs889312 | rs252925 | 0.59 | C5orf35 | 2.30E-08 | Lymphoblastoid | (Nica et al., 2011) |
| rs889312 | rs702691 | 0.60 | C5orf35 | 2.40E-06 | Adipose | (Nica et al., 2011) |
| rs889312 | rs702691 | 0.60 | C5orf35 | 7.10E-09 | Lymphoblastoid | (Nica et al., 2011) |
| rs889312 | rs702691 | 0.60 | C5orf35 | 2.80E-08 | Lymphoblastoid | (Nica et al., 2011) |
| rs889312 | rs832540 | 0.59 | C5orf35 | 2.40E-06 | Adipose | (Nica et al., 2011) |
| rs889312 | rs832540 | 0.59 | C5orf35 | 4.70E-06 | Adipose | (Nica et al., 2011) |
| rs889312 | rs832540 | 0.59 | C5orf35 | 8.70E-10 | Lymphoblastoid | (Nica et al., 2011) |
| rs889312 | rs832540 | 0.59 | C5orf35 | 4.20E-09 | Lymphoblastoid | (Nica et al., 2011) |
| rs889312 | rs832552 | 0.61 | C5orf35 | 5.60E-10 | Lymphoblastoid | (Nica et al., 2011) |
| rs889312 | rs832552 | 0.61 | C5orf35 | 6.90E-08 | Lymphoblastoid | (Nica et al., 2011) |
| rs6504950 | rs17745344 | 0.99 | COX11 | <1e-6 | Adipose | (Grundberg et al., 2012) |
| rs6504950 | rs17745344 | 0.99 | COX11 | <1e-10 | Lymphoblastoid | (Grundberg et al., 2012) |
| rs4808801 | rs2385089 | 0.88 | ELL | <1e-36 | Lymphoblastoid | (Grundberg et al., 2012) |
| rs889312 | rs331499 | 0.56 | C5orf35 | <1e-10 | Adipose | (Grundberg et al., 2012) |
| rs889312 | rs331499 | 0.56 | C5orf35 | <1e-20 | Lymphoblastoid | (Grundberg et al., 2012) |
| rs889312 | rs331499 | 0.56 | C5orf35 | <1e-20 | Skin | (Grundberg et al., 2012) |
| rs11552449 | rs3761936 | 0.99 | BCL2L15 | <1e-10 | Lymphoblastoid | (Grundberg et al., 2012) |
| rs3903072 | rs633800 | 0.78 | BANF1 | <1e-8 | Adipose | (Grundberg et al., 2012) |
| rs3903072 | rs633800 | 0.78 | CTSW | <1e-6 | Lymphoblastoid | (Grundberg et al., 2012) |
| rs3903072 | rs633800 | 0.78 | CTSW | <1e-5 | Skin | (Grundberg et al., 2012) |
| rs2380205 | rs6602322 | 0.53 | ANKRD16 | <1e-6 | Skin | (Grundberg et al., 2012) |
| rs889312 | rs702691 | 0.60 | C5orf35 | <1e-10 | Adipose | (Grundberg et al., 2012) |
| rs889312 | rs702691 | 0.60 | C5orf35 | <1e-15 | Lymphoblastoid | (Grundberg et al., 2012) |
| rs889312 | rs702691 | 0.60 | C5orf35 | <1e-15 | Skin | (Grundberg et al., 2012) |
| rs6504950 | rs7222197 | 1.00 | COX11 | <1e-6 | Adipose | (Grundberg et al., 2012) |
| rs6504950 | rs7222197 | 1.00 | COX11 | <1e-10 | Lymphoblastoid | (Grundberg et al., 2012) |
| rs6504950 | rs7226272 | 0.55 | COX11 | <1e-5 | Lymphoblastoid | (Grundberg et al., 2012) |
| rs889312 | rs832540 | 0.59 | C5orf35 | <1e-12 | Adipose | (Grundberg et al., 2012) |
| rs889312 | rs832540 | 0.59 | C5orf35 | <1e-20 | Lymphoblastoid | (Grundberg et al., 2012) |
| rs889312 | rs832540 | 0.59 | C5orf35 | <1e-20 | Skin | (Grundberg et al., 2012) |
| rs889312 | rs832552 | 0.61 | C5orf35 | <1e-10 | Adipose | (Grundberg et al., 2012) |
| rs889312 | rs832552 | 0.61 | C5orf35 | <1e-15 | Lymphoblastoid | (Grundberg et al., 2012) |
| rs889312 | rs832552 | 0.61 | C5orf35 | <1e-15 | Skin | (Grundberg et al., 2012) |
| rs6504950 | rs9915183 | 1.00 | COX11 | <1e-6 | Adipose | (Grundberg et al., 2012) |
| rs6504950 | rs9915183 | 1.00 | COX11 | <1e-10 | Lymphoblastoid | (Grundberg et al., 2012) |
| index SNP | High LD  SNP | r^2^ | target Gene | eQTL Bayes Factor | Cell type | reference |
| rs889312 | rs1466008 | 0.56 | C5orf35 | 67.6 | lymphoblastoid | (Mangravite et al., in review) |
| rs889312 | rs192249 | 0.59 | C5orf35 | 66.3 | lymphoblastoid | (Mangravite et al., in review) |
| rs889312 | rs194059 | 0.59 | C5orf35 | 65.1 | lymphoblastoid | (Mangravite et al., in review) |
| rs889312 | rs252913 | 0.59 | C5orf35 | 66.8 | lymphoblastoid | (Mangravite et al., in review) |
| rs889312 | rs252914 | 0.59 | C5orf35 | 66.5 | lymphoblastoid | (Mangravite et al., in review) |
| rs889312 | rs252923 | 0.57 | C5orf35 | 67.8 | lymphoblastoid | (Mangravite et al., in review) |
| rs889312 | rs252925 | 0.59 | C5orf35 | 67.5 | lymphoblastoid | (Mangravite et al., in review) |
| rs4245739 | rs2926534 | 0.99 | MDM4 | 74.3 | lymphoblastoid | (Mangravite et al., in review) |
| rs4245739 | rs3014606 | 0.99 | MDM4 | 73.2 | lymphoblastoid | (Mangravite et al., in review) |
| rs889312 | rs331499 | 0.56 | C5orf35 | 67.8 | lymphoblastoid | (Mangravite et al., in review) |
| rs889312 | rs33318 | 0.56 | C5orf35 | 68.0 | lymphoblastoid | (Mangravite et al., in review) |
| rs4245739 | rs4245736 | 0.76 | MDM4 | 72.7 | lymphoblastoid | (Mangravite et al., in review) |
| rs4245739 | rs4245737 | 0.73 | MDM4 | 72.7 | lymphoblastoid | (Mangravite et al., in review) |
| rs4245739 | rs4951075 | 0.73 | MDM4 | 76.3 | lymphoblastoid | (Mangravite et al., in review) |
| rs889312 | rs702691 | 0.60 | C5orf35 | 61.7 | lymphoblastoid | (Mangravite et al., in review) |
| rs889312 | rs832534 | 0.56 | C5orf35 | 67.2 | lymphoblastoid | (Mangravite et al., in review) |
| rs889312 | rs832535 | 0.56 | C5orf35 | 67.4 | lymphoblastoid | (Mangravite et al., in review) |
| rs889312 | rs832536 | 0.55 | C5orf35 | 67.6 | lymphoblastoid | (Mangravite et al., in review) |
| rs889312 | rs832540 | 0.59 | C5orf35 | 62.4 | lymphoblastoid | (Mangravite et al., in review) |
| rs889312 | rs832552 | 0.61 | C5orf35 | 58.8 | lymphoblastoid | (Mangravite et al., in review) |
