# Supplementary material for: Comprehensive Functional Annotation of Seventy-One Breast Cancer Risk Loci
Source: PLoS One. 2013 May 22;8(5):e63925. doi: 10.1371/journal.pone.0063925 (PMC3661550; doi:10.1371/journal.pone.0063925)
Supplement: Table S10 — Breast Cancer Enhancer (BCE) regions used for luciferase assays. (DOC) [file pone.0063925.s016.doc]

Table S10. Breast Cancer Enhancer (BCE) regions used for luciferase assays.

| risk region # | chromosome | # of biofeature | corr.snp.id | bio.feature | index.snp.id | R.squared | nearest.  lincRNA.ID | nearest.  TSS.Gene  Symbol | cloned region name |
| --- | --- | --- | --- | --- | --- | --- | --- | --- | --- |
| 6 | 2p24.1 | 6 | rs12710697 | Enhancer_HMEC_HMMEnhancer | rs12710696 | 0.74 | TCONS_00004704 | MIR4757 | BCE1 |
|  |  | 5 | rs10206829 | Enhancer_HMEC_H3K27Ac | rs12710696 | 0.74 |  |  |  |
|  |  | 5 | rs6731836 | Enhancer_HMEC_H3K9Ac | rs12710696 | 1.00 |  |  |  |
|  |  | 5 | rs6741887 | Enhancer_HMEC_H3K4me1 | rs12710696 | 0.91 |  |  |  |
|  |  | 5 | rs4425044 | Enhancer_HMEC_H3K4me2 | rs12710696 | 0.74 |  |  |  |
|  |  | 4 | rs4322801 | Enhancer_HMEC_H3K9Ac | rs12710696 | 0.74 |  |  |  |
|  |  |  |  | Enhancer_HMEC_H3K4me1 |  |  |  |  |  |
|  |  |  |  | Enhancer_HMEC_FAIRE |  |  |  |  |  |
| 6 | 2p24.1 | 8 | rs4233763 | Enhancer_HMEC_Uw_Rep2_DNaseI | rs12710696 | 0.74 | TCONS_00004704 | MIR4757 | BCE2 |
|  |  | 8 | rs4233764 | Enhancer_HMEC_HMMEnhancer | rs12710696 | 0.74 |  |  |  |
|  |  |  |  | Enhancer_HMEC_H3K9Ac |  |  |  |  |  |
|  |  |  |  | Enhancer_HMEC_H3K4me2 |  |  |  |  |  |
|  |  |  |  | Enhancer_HMEC_H3K4me1 |  |  |  |  |  |
|  |  |  |  | Enhancer_HMEC_Uw_Rep1_DNaseI |  |  |  |  |  |
|  |  |  |  | Enhancer_HMEC_H3K27Ac |  |  |  |  |  |
|  |  |  |  | Enhancer_HMEC_FAIRE |  |  |  |  |  |
| 14 | 3p24.1 | 5 | rs55676236 | Enhancer_HMEC_HMMEnhancer | rs4973768 | 0.57 | TCONS_00005969 | NEK10 | BCE3 |
|  |  |  |  | Enhancer_HMEC_H3K9Ac |  |  |  |  |  |
|  |  |  |  | Enhancer_HMEC_H3K4me2 |  |  |  |  |  |
|  |  |  |  | Enhancer_HMEC_H3K4me1 |  |  |  |  |  |
|  |  |  |  | Enhancer_HMEC_H3K27Ac |  |  |  |  |  |
| 18 | 5p12 | 5 | rs4412123 | Enhancer_HMEC_H3K4me1 | rs4415084 | 0.83 | TCONS_00009394 | MRPS30 | BCE4 |
|  |  |  |  | Enhancer_HMEC_H3K27Ac |  |  |  |  |  |
|  |  |  |  | Enhancer_HMEC_H3K4me2 |  |  |  |  |  |
|  |  |  |  | Enhancer_HMEC_H3K9Ac |  |  |  |  |  |
|  |  |  |  | Enhancer_HMEC_HMMEnhancer |  |  |  |  |  |
| 34 | 8q24.21 | 7 | rs28759353 | Enhancer_HMEC_H3K27Ac | rs13281615 | 0.63 | TCONS_00015171 | POU5F1B | BCE5 |
|  |  | 6 | rs4871782 | Enhancer_HMEC_H3K4me1 | rs13281615 | 0.92 |  |  |  |
|  |  | 5 | rs10087810 | Enhancer_HMEC_H3K4me2 | rs13281615 | 0.82 |  |  |  |
|  |  |  |  | Enhancer_HMEC_H3K9Ac |  |  |  |  |  |
|  |  |  |  | Enhancer_HMEC_HMMEnhancer |  |  |  |  |  |
|  |  |  |  | Enhancer_HMEC_FAIRE |  |  |  |  |  |
|  |  |  |  | Enhancer_HMEC_Uw_Rep1_DNaseI |  |  |  |  |  |
| 42 | 10q22.3 | 5 | rs4980021 | Enhancer_HMEC_H3K4me2 | rs704010 | 0.54 | TCONS_00018541 | ZMIZ1 | BCE6 |
|  |  |  |  | Enhancer_HMEC_H3K9Ac |  |  |  |  |  |
|  |  |  |  | Enhancer_HMEC_Duke_DNaseI |  |  |  |  |  |
|  |  |  |  | Enhancer_HMEC_H3K4me1 |  |  |  |  |  |
|  |  |  |  | Enhancer_HMEC_HMMEnhancer |  |  |  |  |  |
| 50 | 12p11 | 5 | rs813722 | Enhancer_HMEC_H3K4me2 | rs10771399 | 0.74 | TCONS_00020365 | PTHLH | BCE7 |
|  |  |  |  | Enhancer_HMEC_H3K9Ac |  |  |  |  |  |
|  |  |  |  | Enhancer_HMEC_H3K4me1 |  |  |  |  |  |
|  |  |  |  | Enhancer_HMEC_H3K27Ac |  |  |  |  |  |
|  |  |  |  | Enhancer_HMEC_HMMEnhancer |  |  |  |  |  |
| 50 | 12p11 | 7 | rs788463 | Enhancer_HMEC_Uw_Rep2_DNaseI | rs10771399 | 0.93 | TCONS_00020365 | PTHLH | BCE8 |
|  |  |  |  | Enhancer_HMEC_Uw_Rep1_DNaseI |  |  |  |  |  |
|  |  |  |  | Enhancer_HMEC_H3K4me1 |  |  |  |  |  |
|  |  |  |  | Enhancer_HMEC_H3K27Ac |  |  |  |  |  |
|  |  |  |  | Enhancer_HMEC_H3K4me2 |  |  |  |  |  |
|  |  |  |  | Enhancer_HMEC_HMMEnhancer |  |  |  |  |  |
|  |  |  |  | Enhancer_HMEC_FAIRE |  |  |  |  |  |
| 57 | 14q24.1 | 7 | rs17828907 | Enhancer_HMEC_H3K4me2 | rs999737 | 0.82 | TCONS_00022775 | ZFP36L1 | BCE9 |
|  |  |  |  | Enhancer_HMEC_H3K9Ac |  |  |  |  |  |
|  |  |  |  | Enhancer_HMEC_FAIRE |  |  |  |  |  |
|  |  |  |  | Enhancer_HMEC_H3K27Ac |  |  |  |  |  |
|  |  |  |  | Enhancer_HMEC_HMMEnhancer |  |  |  |  |  |
|  |  |  |  | Enhancer_HMEC_H3K4me1 |  |  |  |  |  |
|  |  |  |  | Enhancer_HMEC_Uw_Rep1_DNaseI |  |  |  |  |  |
| 61 | 16q12.2 | 5 | rs11642015 | Enhancer_HMEC_H3K9Ac | rs17817449 | 0.93 | TCONS_00024420 | FTO | BCE10 |
|  |  | 5 | rs1558901 | Enhancer_HMEC_H3K27Ac | rs17817449 | 0.90 |  |  |  |
|  |  | 5 | rs1558902 | Enhancer_HMEC_HMMEnhancer | rs17817449 | 0.93 |  |  |  |
|  |  | 5 | rs62048402 | Enhancer_HMEC_H3K4me1 | rs17817449 | 0.93 |  |  |  |
|  |  | 5 | rs8055197 | Enhancer_HMEC_H3K4me2 | rs17817449 | 0.62 |  |  |  |
| 61 | 16q12.2 | 6 | rs3751812 | Enhancer_HMEC_H3K4me1 | rs17817449 | 0.99 | TCONS_00024420 | FTO | BCE11 |
|  |  | 5 | rs3751813 | Enhancer_HMEC_H3K9Ac | rs17817449 | 0.57 |  |  |  |
|  |  | 5 | rs11075987 | Enhancer_HMEC_H3K27Ac | rs17817449 | 0.65 |  |  |  |
|  |  | 5 | rs3751814 | Enhancer_HMEC_HMMEnhancer | rs17817449 | 0.99 |  |  |  |
|  |  | 5 | rs56313538 | Enhancer_HMEC_H3K4me2 | rs17817449 | 0.99 |  |  |  |
|  |  | 5 | rs9931900 | Enhancer_HMEC_FAIRE | rs17817449 | 0.95 |  |  |  |
|  |  | 5 | rs9933509 |  | rs17817449 | 0.96 |  |  |  |
