# Supplementary material for: Comprehensive Functional Annotation of Seventy-One Breast Cancer Risk Loci
Source: PLoS One. 2013 May 22;8(5):e63925. doi: 10.1371/journal.pone.0063925 (PMC3661550; doi:10.1371/journal.pone.0063925)
Supplement: Table S11 — Oligonucleotide sequences used for cloning and qPCR. (DOC) [file pone.0063925.s017.doc]

Table S11. Oligonucleotide sequences used for cloning and qPCR.

| **Region name** | **Forward primers (5'-3')** | **Reverse primers (5'-3')** | **Cloned regions** |
| --- | --- | --- | --- |
| BCE1 | TTGGGATATTGGAGGAGCTG | TGAATGCTGCACGACTTACC | chr2:19319749-19322150 |
| BCE2 | AAAGTCCTGTCACCCCACAG | CCATCAGATCCAACCCATTT | chr2:19323777-19324820 |
| BCE3 | TTTTCAAGACAATTAATAAGCCCATAA | TTGCAATCTGTTGTGGGACA | chr3:27391058-27392178 |
| BCE4 | GATACTGGCAAAAGCCCTGA | CCTCTCAAATTGTGAAGATTGG | chr5:44875448-44876553 |
| BCE5 | GGGGCATAGAATCAGTGGAGGG | CTGGGGGTGAGGAAGCTAACTC | chr8:128351899-128353121 |
| BCE6 | CTCAGGCCTTACCGATACCA | TCAAGGTCCTGAGCCAGTCT | chr10:80833975-80835085 |
| BCE7 | AACTCATTTTCTCAGTACCTCAGTCA | TGGGGAAAATTCAGAGTCCA | chr12:28151557-28152263 |
| BCE8 | CATCTCTAAAAATGTCTTGGCTACC | TGGTGTTACCCAAATCCAAAA | chr12:28152319-28152861 |
| BCE9 | ACCAGACAGCCTCTCAGGAA | ATGGAACCTGGTGCTCAAAG | chr14:69010612-69011886 |
| BCE10 | CTTTGGAGCCTGGCTTTATG | TTTTCCCCTTCTTGCT | chr16:53801475-53803839 |
| BCE11 | TGGTTGATCATCACCTCACC | GCTGAGTGAGGTCCAAATGC | chr16:53817841-53818941 |
| CT1 | GGGGTACCCCAAGTGGAACCAACTGACA | GGGGTACCGGCCAAAAGAAAATGGCATA | chr8:128497870-128499560 |
| CT2 | GGGGTACCGCATGCATTAGGGGAGAAAA | GGGGTACCGTAGCTCACAGCCGAGATCC | chr8:128512424-128514005 |
| BCE5-1 | TGAGATCATAAGGTAAAAGGGGACA | TCTTGGTTTTTCTCATATTTTTCTGG | chr8:128351124-128351272 |
| BCE5-2 | CCAATCTCCTGCAGAGGTGCT | GACTTGAGCCCAGGTCACCA | chr8:128351512-128351662 |
| BCE5-3 | TGCAAGTGGAATGTACTGAAATCAA | TCTGGCCCTTTAGCTCTTTGG | chr8:128352446-128352595 |
| BCE5-4 | TGAATAACCTGGGTCCCAAA | CCCAGAAGAACTTGGCATAGAGG | chr8:128352823-128353029 |
| BCE5-5 | TCACCAATCACAGGGGAAGC | CACTGGAATTCACAATAAGACATGAA | chr8:128353161-128353287 |
