# Supplementary material for: Comprehensive Functional Annotation of Seventy-One Breast Cancer Risk Loci
Source: PLoS One. 2013 May 22;8(5):e63925. doi: 10.1371/journal.pone.0063925 (PMC3661550; doi:10.1371/journal.pone.0063925)
Supplement: Table S13 — Top 18 TF motifs for high LD SNPs in enhancers. (DOC) [file pone.0063925.s019.doc]

Table S13. Top 18 TF motifs for high LD SNPs in enhancers

| motif_name | number of SNPs within each TF motif's RE |
| --- | --- |
| TAL1 | 28 |
| Eomes | 26 |
| Foxp1 | 24 |
| SP1 | 20 |
| Pou2f1 | 19 |
| MEF2A | 18 |
| TBX5 | 18 |
| E2A | 13 |
| EWSR1-FLI1 | 13 |
| AR | 12 |
| CRX | 12 |
| Egr1 | 11 |
| Nkx2.5 | 11 |
| NKX3-1 | 11 |
| HNF1A | 10 |
| IRF1 | 10 |
| Olig2 | 10 |
| Zfx | 10 |
