# Supplementary material for: Comprehensive Functional Annotation of Seventy-One Breast Cancer Risk Loci
Source: PLoS One. 2013 May 22;8(5):e63925. doi: 10.1371/journal.pone.0063925 (PMC3661550; doi:10.1371/journal.pone.0063925)
Supplement: Table S14 — eQTL analyses on high LD SNPs in enhancers. (DOC) [file pone.0063925.s020.doc]

Table S14. eQTL analyses on high LD SNPs in enhancers

| index SNP | High LD  SNP | R^2^ | target Gene | eQTL  P-value | Cell type | reference |
| --- | --- | --- | --- | --- | --- | --- |
| rs3760982 | rs10421887 | 1.00 | KCNN4 | 1.08E-97 | Monocytes | (Zeller et al., 2010) |
| rs889312 | rs11960484 | 0.62 | MGC33648 | 1.05E-136 | Monocytes | (Zeller et al., 2010) |
| rs4808801 | rs1469412 | 0.51 | ELL | 1.47E-20 | Monocytes | (Zeller et al., 2010) |
| rs4245739 | rs2369244 | 0.72 | MDM4 | 1.69E-19 | Monocytes | (Zeller et al., 2010) |
| rs889312 | rs252905 | 0.61 | MGC33648 | 9.18E-138 | Monocytes | (Zeller et al., 2010) |
| rs6504950 | rs2628305 | 0.87 | COX11 | 1.28E-16 | Monocytes | (Zeller et al., 2010) |
| rs3760982 | rs3760983 | 1.00 | KCNN4 | 1.04E-99 | Monocytes | (Zeller et al., 2010) |
| rs3760982 | rs3786956 | 0.51 | KCNN4 | 6.56E-47 | Monocytes | (Zeller et al., 2010) |
| rs4245739 | rs4252725 | 0.78 | MDM4 | 2.40E-21 | Monocytes | (Zeller et al., 2010) |
| rs4245739 | rs4951393 | 0.72 | MDM4 | 2.11E-19 | Monocytes | (Zeller et al., 2010) |
| rs4245739 | rs4951409 | 0.61 | MDM4 | 4.19E-17 | Monocytes | (Zeller et al., 2010) |
| rs4245739 | rs6679717 | 0.61 | MDM4 | 6.38E-17 | Monocytes | (Zeller et al., 2010) |
| rs3903072 | rs677029 | 0.53 | BANF1 | 9.15E-16 | Monocytes | (Zeller et al., 2010) |
| rs4245739 | rs7532236 | 0.61 | MDM4 | 3.80E-17 | Monocytes | (Zeller et al., 2010) |
| rs2046210 | rs7740686 | 0.91 | C6orf97 | 4.81E-14 | Monocytes | (Zeller et al., 2010) |
| rs6504950 | rs9904377 | 0.71 | COX11 | 4.32E-23 | Monocytes | (Zeller et al., 2010) |
| rs4245739 | rs11240751 | 0.73 | MDM4 | 7.40E-06 | Lymphoblastoid | (Stranger et al., 2012) |
| rs4245739 | rs11240751 | 0.73 | MDM4 | 1.10E-06 | Lymphoblastoid | (Stranger et al., 2012) |
| rs889312 | rs11960484 | 0.62 | C5orf35 | 1.80E-06 | Lymphoblastoid | (Stranger et al., 2012) |
| rs4245739 | rs2369244 | 0.72 | MDM4 | 1.60E-07 | Lymphoblastoid | (Stranger et al., 2012) |
| rs4245739 | rs4252725 | 0.78 | MDM4 | 1.60E-07 | Lymphoblastoid | (Stranger et al., 2012) |
| rs4245739 | rs4951389 | 0.74 | MDM4 | 9.00E-06 | Lymphoblastoid | (Stranger et al., 2012) |
| rs4245739 | rs4951389 | 0.74 | MDM4 | 1.60E-07 | Lymphoblastoid | (Stranger et al., 2012) |
| rs4245739 | rs4951393 | 0.72 | MDM4 | 1.20E-06 | Lymphoblastoid | (Stranger et al., 2012) |
| rs4245739 | rs4951393 | 0.72 | MDM4 | 1.60E-07 | Lymphoblastoid | (Stranger et al., 2012) |
| rs4245739 | rs4951393 | 0.72 | MDM4 | 3.50E-06 | Lymphoblastoid | (Stranger et al., 2012) |
| rs4245739 | rs4951409 | 0.61 | MDM4 | 3.20E-07 | Lymphoblastoid | (Stranger et al., 2012) |
| rs4245739 | rs6679717 | 0.61 | MDM4 | 5.30E-07 | Lymphoblastoid | (Stranger et al., 2012) |
| rs4808801 | rs10405636 | 0.90 | SSBP4 | 3.41E-10 | Lymphoblastoid | (Pickrell et al., 2010) |
| rs4808801 | rs11670392 | 0.51 | ELL | 4.60E-06 | Lymphoblastoid | (Nica et al., 2011) |
| rs889312 | rs11960484 | 0.62 | C5orf35 | 6.20E-08 | Lymphoblastoid | (Nica et al., 2011) |
| rs889312 | rs11960484 | 0.62 | C5orf35 | 4.20E-10 | Lymphoblastoid | (Nica et al., 2011) |
| rs3903072 | rs637571 | 0.59 | BANF1 | 6.00E-06 | Adipose | (Nica et al., 2011) |
| rs4808801 | rs1469412 | 0.51 | UBA52 | 1.03E-09 | Cortex | (Myers et al., 2007) |
| rs3760982 | rs3760983 | 1.00 | FLJ30469 | 4.68E-18 | Cortex | (Myers et al., 2007) |
| rs3760982 | rs3786956 | 0.51 | FLJ30469 | 2.22E-21 | Cortex | (Myers et al., 2007) |
| rs4808801 | rs10164319 | 0.99 | ELL | <1E-35 | Lympoblast | (Grundberg et al., 2012) |
| rs4808801 | rs10405636 | 0.90 | ELL | <1E-38 | Lympoblast | (Grundberg et al., 2012) |
| rs4808801 | rs10420384 | 0.51 | ELL | 1.00E-40 | Lympoblast | (Grundberg et al., 2012) |
| rs4808801 | rs10420384 | 0.51 | UBA52 | <1E-6 | Skin | (Grundberg et al., 2012) |
| rs4808801 | rs10426768 | 0.51 | ELL | <1E-35 | Lympoblast | (Grundberg et al., 2012) |
| rs4808801 | rs10426768 | 0.51 | UBA52 | <1E-6 | Skin | (Grundberg et al., 2012) |
| rs3903072 | rs11227311 | 0.90 | CTSW | <1E-5 | Skin | (Grundberg et al., 2012) |
| rs3903072 | rs11227311 | 0.90 | BANF1 | <1E-5 | Adipose | (Grundberg et al., 2012) |
| rs4245739 | rs11240751 | 0.73 | MDM4 | <1E-6 | Skin | (Grundberg et al., 2012) |
| rs4245739 | rs11240751 | 0.73 | MDM4 | <1E-19 | Lympoblast | (Grundberg et al., 2012) |
| rs3903072 | rs1151523 | 0.57 | BANF1 | <1E-11 | Adipose | (Grundberg et al., 2012) |
| rs12493607 | rs1155705 | 0.99 | TGFBR2 | <1E-5 | Lympoblast | (Grundberg et al., 2012) |
| rs12493607 | rs1155708 | 0.99 | TGFBR2 | <1E-5 | Lympoblast | (Grundberg et al., 2012) |
| rs3760982 | rs11665924 | 1.00 | ZNF404 | <1E-5 | Lympoblast | (Grundberg et al., 2012) |
| rs4808801 | rs11670392 | 0.51 | UBA52 | <1E-6 | Skin | (Grundberg et al., 2012) |
| rs4808801 | rs11670392 | 0.51 | ELL | <1E-35 | Lympoblast | (Grundberg et al., 2012) |
| rs889312 | rs11960484 | 0.62 | C5orf35 | <1E-19 | Lympoblast | (Grundberg et al., 2012) |
| rs889312 | rs11960484 | 0.62 | C5orf35 | <1E-10 | Adipose | (Grundberg et al., 2012) |
| rs889312 | rs11960484 | 0.62 | C5orf35 | <1E-15 | Skin | (Grundberg et al., 2012) |
| rs616488 | rs12129512 | 0.52 | PEX14 | <1E-10 | Adipose | (Grundberg et al., 2012) |
| rs12493607 | rs12495646 | 1.00 | TGFBR2 | <1E-5 | Lympoblast | (Grundberg et al., 2012) |
| rs12493607 | rs13061018 | 0.87 | TGFBR2 | <1E-5 | Lympoblast | (Grundberg et al., 2012) |
| rs12493607 | rs13081020 | 0.99 | TGFBR2 | <1E-5 | Lympoblast | (Grundberg et al., 2012) |
| rs12493607 | rs13083158 | 0.87 | TGFBR2 | <1E-5 | Lympoblast | (Grundberg et al., 2012) |
| rs3760982 | rs1386502 | 0.60 | ZNF404 | <1E-5 | Lympoblast | (Grundberg et al., 2012) |
| rs4808801 | rs1469412 | 0.51 | ELL | <1E-35 | Lympoblast | (Grundberg et al., 2012) |
| rs4808801 | rs1469412 | 0.51 | UBA52 | <1E-6 | Skin | (Grundberg et al., 2012) |
| rs4808801 | rs1560118 | 0.99 | ELL | <1E-35 | Lympoblast | (Grundberg et al., 2012) |
| rs616488 | rs2026792 | 0.73 | PEX14 | <1E-11 | Adipose | (Grundberg et al., 2012) |
| rs4808801 | rs2278238 | 0.95 | ELL | <1E-35 | Lympoblast | (Grundberg et al., 2012) |
| rs4245739 | rs2369244 | 0.72 | MDM4 | <1E-17 | Lympoblast | (Grundberg et al., 2012) |
| rs4245739 | rs2369244 | 0.72 | MDM4 | <1E-6 | Skin | (Grundberg et al., 2012) |
| rs889312 | rs252905 | 0.61 | C5orf35 | <1E-15 | Skin | (Grundberg et al., 2012) |
| rs889312 | rs252905 | 0.61 | C5orf35 | <1E-18 | Lympoblast | (Grundberg et al., 2012) |
| rs889312 | rs252905 | 0.61 | C5orf35 | <1E-10 | Adipose | (Grundberg et al., 2012) |
| rs6504950 | rs2628305 | 0.87 | COX11 | <1E-6 | Adipose | (Grundberg et al., 2012) |
| rs6504950 | rs2628305 | 0.87 | COX11 | <1E-7 | Lympoblast | (Grundberg et al., 2012) |
| rs4808801 | rs271621 | 0.95 | ELL | <1E-35 | Lympoblast | (Grundberg et al., 2012) |
| rs889312 | rs3099460 | 0.62 | C5orf35 | <1E-15 | Skin | (Grundberg et al., 2012) |
| rs889312 | rs3099460 | 0.62 | C5orf35 | <1E-10 | Adipose | (Grundberg et al., 2012) |
| rs889312 | rs3099460 | 0.62 | C5orf35 | <1E-18 | Lympoblast | (Grundberg et al., 2012) |
| rs889312 | rs33321 | 0.58 | C5orf35 | <1E-12 | Adipose | (Grundberg et al., 2012) |
| rs889312 | rs33321 | 0.58 | C5orf35 | <1E-23 | Lympoblast | (Grundberg et al., 2012) |
| rs889312 | rs33321 | 0.58 | C5orf35 | <1E-20 | Skin | (Grundberg et al., 2012) |
| rs3760982 | rs3760983 | 1.00 | ZNF404 | <1E-5 | Lympoblast | (Grundberg et al., 2012) |
| rs3760982 | rs3786956 | 0.51 | ZNF404 | <1E-5 | Lympoblast | (Grundberg et al., 2012) |
| rs4245739 | rs4252725 | 0.78 | MDM4 | <1E-6 | Skin | (Grundberg et al., 2012) |
| rs4245739 | rs4252725 | 0.78 | MDM4 | <1E-15 | Lympoblast | (Grundberg et al., 2012) |
| rs3760982 | rs4803659 | 0.60 | ZNF404 | <1E-5 | Lympoblast | (Grundberg et al., 2012) |
| rs4245739 | rs4951389 | 0.74 | MDM4 | <1E-18 | Lympoblast | (Grundberg et al., 2012) |
| rs4245739 | rs4951393 | 0.72 | MDM4 | <1E-18 | Lympoblast | (Grundberg et al., 2012) |
| rs4245739 | rs4951409 | 0.61 | MDM4 | <1E-15 | Lympoblast | (Grundberg et al., 2012) |
| rs616488 | rs596537 | 0.71 | PEX14 | <1E-12 | Adipose | (Grundberg et al., 2012) |
| rs616488 | rs607941 | 0.71 | PEX14 | <1E-12 | Adipose | (Grundberg et al., 2012) |
| rs616488 | rs620405 | 0.71 | PEX14 | <1E-12 | Adipose | (Grundberg et al., 2012) |
| rs616488 | rs622623 | 0.71 | PEX14 | <1E-12 | Adipose | (Grundberg et al., 2012) |
| rs3903072 | rs634534 | 0.58 | BANF1 | <1E-12 | Adipose | (Grundberg et al., 2012) |
| rs616488 | rs636291 | 0.60 | PEX14 | <1E-12 | Adipose | (Grundberg et al., 2012) |
| rs616488 | rs648324 | 0.71 | PEX14 | <1E-12 | Adipose | (Grundberg et al., 2012) |
| rs616488 | rs648399 | 0.71 | PEX14 | <1E-12 | Adipose | (Grundberg et al., 2012) |
| rs616488 | rs660725 | 0.71 | PEX14 | <1E-12 | Adipose | (Grundberg et al., 2012) |
| rs616488 | rs662064 | 0.70 | PEX14 | <1E-12 | Adipose | (Grundberg et al., 2012) |
| rs3903072 | rs677029 | 0.53 | BANF1 | <1E-13 | Adipose | (Grundberg et al., 2012) |
| rs616488 | rs685746 | 0.62 | PEX14 | <1E-13 | Adipose | (Grundberg et al., 2012) |
| index SNP | High LD  SNP | R^2^ | target Gene | eQTL Posterior Probability | Cell type | reference |
| rs4808801 | rs10442 | 0.94 | SSBP4 | 0.187 | Lymphoblastoid | (Veyrieras et al., 2008) |
| rs4245739 | rs2369244 | 0.72 | MDM4 | 0.014 | Lymphoblastoid | (Veyrieras et al., 2008) |
| rs4245739 | rs4252725 | 0.78 | MDM4 | 0.045 | Lymphoblastoid | (Veyrieras et al., 2008) |
| rs4245739 | rs6679717 | 0.61 | MDM4 | 6.37E-08 | Lymohoblastoid | (Veyrieras et al., 2008) |
| index SNP | High LD  SNP | R^2^ | target Gene | eQTL Bayes Factor | Cell type | reference |
| rs4245739 | rs10900600 | 0.62 | MDM4 | 58.17 | lymphoblastoid | (Mangravite et al., in review) |
| rs4245739 | rs11240751 | 0.73 | MDM4 | 75.88 | lymphoblastoid | (Mangravite et al., in review) |
| rs4245739 | rs11240754 | 0.78 | MDM4 | 70.87 | lymphoblastoid | (Mangravite et al., in review) |
| rs4245739 | rs11240755 | 0.77 | MDM4 | 71.22 | lymphoblastoid | (Mangravite et al., in review) |
| rs889312 | rs11960484 | 0.62 | C5orf35 | 59.45 | lymphoblastoid | (Mangravite et al., in review) |
| rs4245739 | rs2369244 | 0.72 | MDM4 | 70.13 | lymphoblastoid | (Mangravite et al., in review) |
| rs889312 | rs252905 | 0.61 | C5orf35 | 61.32 | lymphoblastoid | (Mangravite et al., in review) |
| rs889312 | rs2662040 | 0.56 | C5orf35 | 64.87 | lymphoblastoid | (Mangravite et al., in review) |
| rs889312 | rs3099460 | 0.62 | C5orf35 | 62.92 | lymphoblastoid | (Mangravite et al., in review) |
| rs889312 | rs33319 | 0.56 | C5orf35 | 67.93 | lymphoblastoid | (Mangravite et al., in review) |
| rs889312 | rs33321 | 0.58 | C5orf35 | 67.89 | lymphoblastoid | (Mangravite et al., in review) |
| rs4245739 | rs35270244 | 0.73 | MDM4 | 69.37 | lymphoblastoid | (Mangravite et al., in review) |
| rs4245739 | rs4252725 | 0.78 | MDM4 | 69.66 | lymphoblastoid | (Mangravite et al., in review) |
| rs4245739 | rs4951389 | 0.74 | MDM4 | 67.55 | lymphoblastoid | (Mangravite et al., in review) |
| rs4245739 | rs4951393 | 0.72 | MDM4 | 72.88 | lymphoblastoid | (Mangravite et al., in review) |
| rs4245739 | rs4951400 | 0.72 | MDM4 | 69.43 | lymphoblastoid | (Mangravite et al., in review) |
| rs4245739 | rs4951409 | 0.61 | MDM4 | 55.06 | lymphoblastoid | (Mangravite et al., in review) |
| rs4245739 | rs6594015 | 0.91 | MDM4 | 74.96 | lymphoblastoid | (Mangravite et al., in review) |
| rs4245739 | rs6679311 | 0.80 | MDM4 | 51.93 | lymphoblastoid | (Mangravite et al., in review) |
| rs4245739 | rs6679717 | 0.61 | MDM4 | 56.33 | lymphoblastoid | (Mangravite et al., in review) |
| rs4245739 | rs7519417 | 0.90 | MDM4 | 69.51 | lymphoblastoid | (Mangravite et al., in review) |
| rs4245739 | rs7532236 | 0.61 | MDM4 | 55.52 | lymphoblastoid | (Mangravite et al., in review) |
| rs889312 | rs866223 | 0.62 | C5orf35 | 61.62 | lymphoblastoid | (Mangravite et al., in review) |
